# Biobased adhesive hydrogels for wound management and tissue repair: From materials to advanced applications

**DOI:** 10.1063/5.0312916

**Published:** 2026-02-23

**Authors:** Dongyang Miao, Yige Gao, Bowen Shi, Wen Zhou, Dong Wang, Jichao Zhang, Jun Song, Miao Cui, Shuo Shi

**Affiliations:** 1 State Key Laboratory for the Development and Utilization of Forest Food Resources, Joint Laboratory of Advanced Biomedical Materials (NFU-UGent), Jiangsu Co-Innovation Center of Efficient Processing and Utilization of Forest Resources, Nanjing Forestry University, Nanjing 210037, China; 2 Jiangsu Engineering Research Center for Digital Textile Inkjet Printing, Key Laboratory of Eco-Textile, Jiangnan University, Ministry of Education, Wuxi, Jiangsu 214122, China; 3 College of Textile and Clothing, Yancheng Institute of Technology, Yancheng 224051, People's Republic of China; 4 Department of Genetics, Stanford University School of Medicine, Stanford, California 94305, USA; 5 School of Fashion and Textiles, The Hong Kong Polytechnic University, Kowloon, Hong Kong, People's Republic of China

## Abstract

Effective tissue adhesion under wet and dynamic physiological conditions remains a major challenge in wound management and tissue repair. Conventional tissue adhesives often suffer from limited biocompatibility, inadequate wet adhesion, or lack of degradability, restricting their broader clinical use. In recent years, biobased adhesive hydrogels have emerged as promising alternatives owing to their intrinsic biocompatibility, tunable mechanical properties, and capability to achieve robust adhesion in complex biological environments. In addition to being passive sealants, recent developments have made these materials smartly responsive to various stimuli (pH, temperature, light, magnetic field, ultrasound), achieving multifunctional and on-demand treatment performance. This review summarizes recent advances in biobased adhesive hydrogels, with emphasis on material design strategies, interfacial adhesion mechanisms, evaluation methodologies, and representative biomedical applications. Particular attention is given to how biobased materials address the challenges of wet-tissue adhesion, mechanical mismatch, and dynamic tissue interfaces. Finally, current limitations and future development directions are discussed, including translational challenges, minimally invasive applications, and the integration of multifunctionality with clinical practicality. This review aims to provide guidance for the rational design and clinical translation of next-generation biobased adhesive hydrogels.

## I. INTRODUCTION

Uncontrolled bleeding and inefficient wound closure are key issues in trauma care, surgery, and chronic wound management. Chronic wounds such as diabetic ulcers and pressure ulcers place a heavy burden on the medical system.^1–3^ Traditional wound closure techniques, such as suturing and staples, have good mechanical effects, but they can cause secondary tissue damage, cannot fit irregular wounds, and have poor adhesion in dynamic and moist situations.^4–6^ They are not suitable for the situation where fragile tissues need strong and compliant adhesion.^7,8^ These limitations have driven the development of bioadhesive materials to achieve rapid sealing of the bio-environment, prevent infection, and promote tissue regeneration. Ideal biological adhesives have several strict requirements.^9^ It can form a firm and long-lasting wet adhesion on wet, moving, and complex tissue surfaces and seal wounds and prevent body fluid leakage. It also provides a moist and bioactive microenvironment to promote cell proliferation and extracellular matrix deposition. In addition, excellent biocompatibility and controllable degradation are the key to reducing inflammation, preventing secondary injury, and promoting tissue repair. Finally, its mechanical properties match those of natural tissues to avoid damage caused by stress shielding and friction in high-motion organs such as joints and the heart. Under wet and dynamic physiological conditions, it presents a state of variable stability with long-term high adhesion force and toughness.^10^ Therefore, understanding the adhesion mechanism and material design principles is the key to overcoming these obstacles.

From traditional fibrin and cyanoacrylate glues to modern hydrogel systems, the development of biological adhesives has gone through several stages.^11–13^ The first-generation fibrin adhesives, such as Tisseel^®^ and Evicel^®^, use the human coagulation cascade to form a fibrin mesh to physiologically close wounds.^14^ Although biocompatible, these systems have insufficient bonding strength and poor moisture resistance, so they are only applied in low-pressure and auxiliary sealing situations. The increased dependence on blood components increases immunogenicity and infection risk. The second-generation cyanoacrylate adhesives rapidly polymerize under tissue moisture and can bind to biological surfaces.^12^ They have the characteristics of high adhesion and rapid curing, but they will slowly release toxic by-products such as formaldehyde, generate heat, and become brittle-not suitable for soft or wet tissues. Therefore, cyanoacrylate glues are only used for external skin closure and emergency wound sealing. To overcome these limitations, hydrogel-based adhesives are a promising new type of bioadhesive.^15,16^ Hydrogels have soft and hydrated polymer networks that can interact strongly with tissues. Commercial products such as DuraSeal™ and CoSeal™ use N-hydroxysuccinimide (NHS) ester-mediated covalent cross-linking between polymer backbones and histamines to achieve durable wet adhesion.^17^ Currently available adhesive hydrogels can be broadly classified into synthetic and biobased adhesive hydrogels (Table I). Synthetic adhesive hydrogels offer tunable mechanical properties and strong adhesion but often rely on non-degradable components or reactive chemistries that may raise biocompatibility concerns. Commercial products are clinically mature and easy to apply; however, they typically suffer from limited wet adhesion, weak mechanical robustness, or insufficient adaptability to dynamic tissues. In contrast, biobased adhesive hydrogels, derived from natural polymers or biomolecules, inherently exhibit superior biocompatibility, degradability, and biointeractivity. These materials provide a versatile platform for addressing wet-tissue adhesion challenges while minimizing foreign-body responses. As such, biobased adhesive hydrogels represent a promising direction for next-generation wound management and tissue repair, motivating the focus of this review.

**TABLE I.** Comparison between synthetic tissue adhesives and biobased adhesive hydrogels.

Aspect	Synthetic tissue adhesives	Biobased adhesive hydrogels	Ref.
Adhesion mechanism	Rapid curing; often irreversible covalent chemistry and/or physical sealing	Multimodal junctions (covalent, dynamic covalent, interlocking) enabling wet-interface bonding	35
Performance in wet environments	Moderate; interfacial water or blood can reduce effective contact	Generally excellent; engineered for wet adhesion via hydration-layer management	36
Mechanical compliance	Tunable; mismatch risk on moving soft tissues	Typically compliant; easier to match dynamic tissues	37
Degradability	Non-degradable or slowly degradable	Commonly biodegradable; degradation can be matched to healing timeline	38
Biocompatibility	Poor; possible concerns regarding toxicity and chronic foreign-body response	Generally excellent; functional crosslinkers still require biosafety validation	39
Manufacturability	Mature industrial processes	Increasingly scalable; but standardization remains challenging	40

Biobased hydrogels based on natural polysaccharides and proteins have attracted increasing attention because of their biocompatibility, degradability, and properties similar to the extracellular matrix.^18–20^ They have abundant functional groups (–OH, –NH_2_, –COOH) and can be chemically modified to form covalent and non-covalent bonds with tissues.^21,22^ For example, chitosan (CS) has a positive charge, natural hemostatic and antibacterial properties, and can promote red blood cell aggregation and platelet activation.^23,24^ Gelatin and collagen have bioactive Arg–Gly–Asp (RGD) sequences for cell adhesion and migration, and physical cross-linking helps to enhance elasticity and structural integrity.^25^ Hyaluronic acid (HA) supports hydration and cell signaling, and its modification chemistry enables tunable cross-linking and bioactivity.^26,27^ Alginate forms ionically cross-linked hydrogels based on divalent cations such as Ca^2+^ and can have the characteristics of rapid gelation and mechanical stability.^28^ Methylation and oxidation can achieve adjustable cross-linking and biological activity effects in alginate hydrogels. Cellulose and bacterial cellulose have the characteristics of high strength and hydrophilicity and are the reinforcing skeletons in composite hydrogels.^29,30^ Oxidized polysaccharides contain aldehyde groups and can form Schiff base bonds with amines in tissues, which greatly enhances the interfacial adhesion when wet. Recent research progress shows that biopolymers modified with catechol and phenylboronic acid can form dynamic and reversible tissue adhesion, achieving strong adhesion and non-invasive separation.^31–33^ Additionally, these hydrogels can encapsulate bioactive agents, such as growth factors, antimicrobial peptides, and nanoparticles, to accelerate healing and prevent infection.^34^ Despite significant progress in tissue sealants (primarily for leakage prevention) and wound closure technologies, achieving reliable adhesion on wet and dynamically moving tissues remains a fundamental challenge. The presence of interfacial water can weaken adhesive bonding by screening intermolecular interactions, while continuous tissue deformation, blood flow, and physiological motion further compromise adhesion stability. In addition, mechanical mismatch between adhesive materials and soft tissues often leads to stress concentration, interfacial failure, or secondary tissue damage. These challenges highlight the urgent need for adhesive systems that can function effectively under complex biological conditions.

**FIG. 1.**
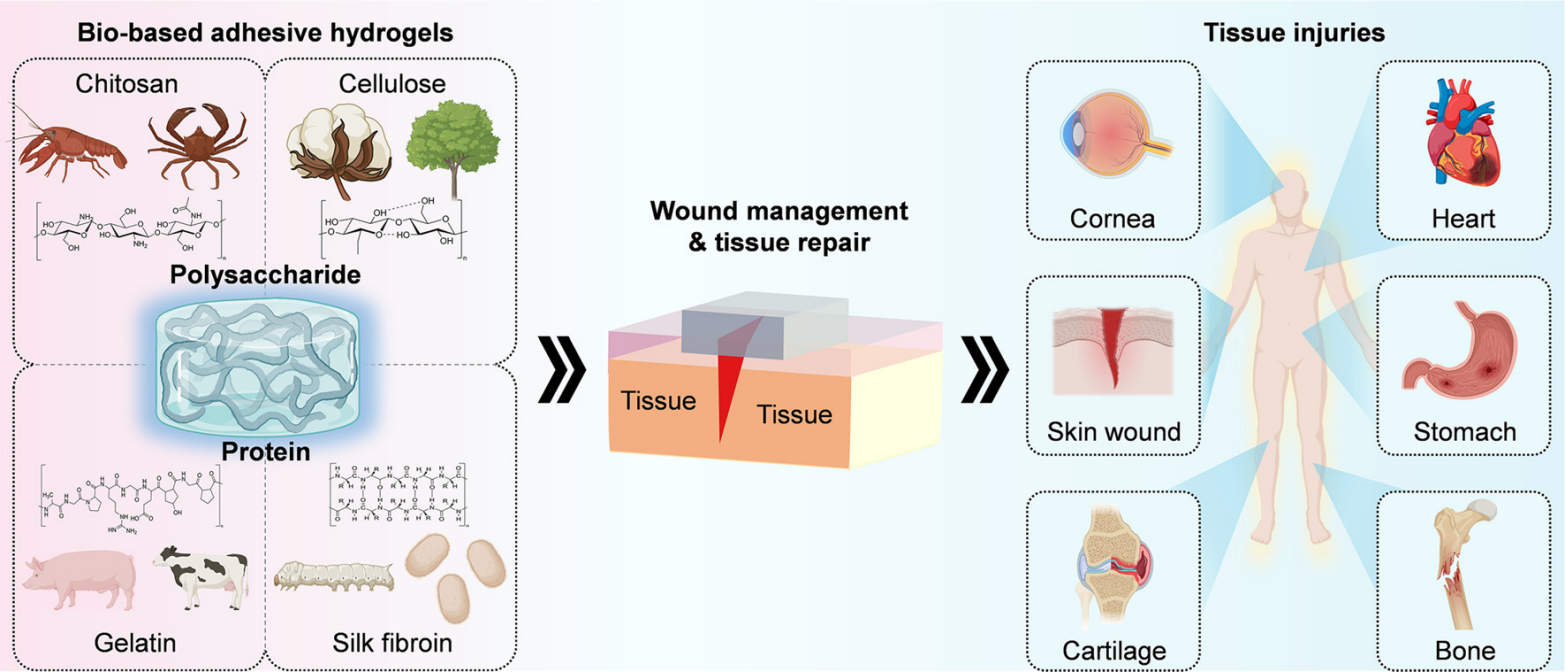
Typical sources of biobased adhesive hydrogels, such as chitosan, cellulose, gelatin, and silk fibroin. Applied in wound management and tissue repair, for example, skin wounds, cardiac injury, gastrointestinal mucosa, cornea, cartilage, and bone defects. Wounds are caused by accidental trauma, surgical incisions, pathological conditions, and adhesive hydrogels can perform rapid or continuous wound management. Created with Biorender.com.

This review provides a comprehensive and mechanism-oriented overview of the latest biobased hydrogels for wound management and tissue repair (Fig. 1). First, the physicochemical mechanisms of interfacial adhesion are expounded, and it is stated that covalent bonds, hydrogen bonds, electrostatic attraction, and mechanical interlocking jointly contribute to strong wet adhesion. Focus is placed on natural polymers and chemical modification strategies to enhance adhesion strength, mechanical properties, and biological functions. The subsequent sections explore the key roles of adhesive hydrogels in two key biomedical applications and evaluation methods-wound sealing and rapid hemostasis, as well as tissue repair and regeneration, emphasizing preformed and *in situ* crosslinkable systems. Finally, current challenges are discussed, such as the trade-off between adhesion strength and biocompatibility, and adaptation to dynamic tissue environments. This review, starting from the aspects of mechanism and application, aims to develop a roadmap for designing the next generation of biobased adhesives, connecting laboratory innovations with clinical translation.

## II. TISSUE ADHESION MECHANISMS

Under wet or dynamic conditions, biobased hydrogels adhere to biological tissues, which is related to interfacial chemistry, reversible bond kinetics, physical entanglement, mechanical interlocking, and water layer mitigation. A framework decomposes the total interfacial adhesion force into covalent bonding energy, reversible non-covalent energy dissipation and self-healing energy, and physical or topological energy dissipation brought about by chain entanglement and mechanical interlocking. This review focuses on the main molecular and mesoscopic mechanisms: first, it describes covalent or dynamic covalent bonds used for strong tissue-hydrogel binding; then, it talks about reversible interactions used for flexibility and toughness; finally, it elaborates on physical and geometric anchoring used for adhesion. It also discusses water management applications, such as interfacial water replacement and isolation in hydrogel networks, so as to stabilize the contact under hydrated conditions.

### A. Physicochemical basis of interfacial adhesion

#### 1. Covalent bonding

**FIG. 2.**
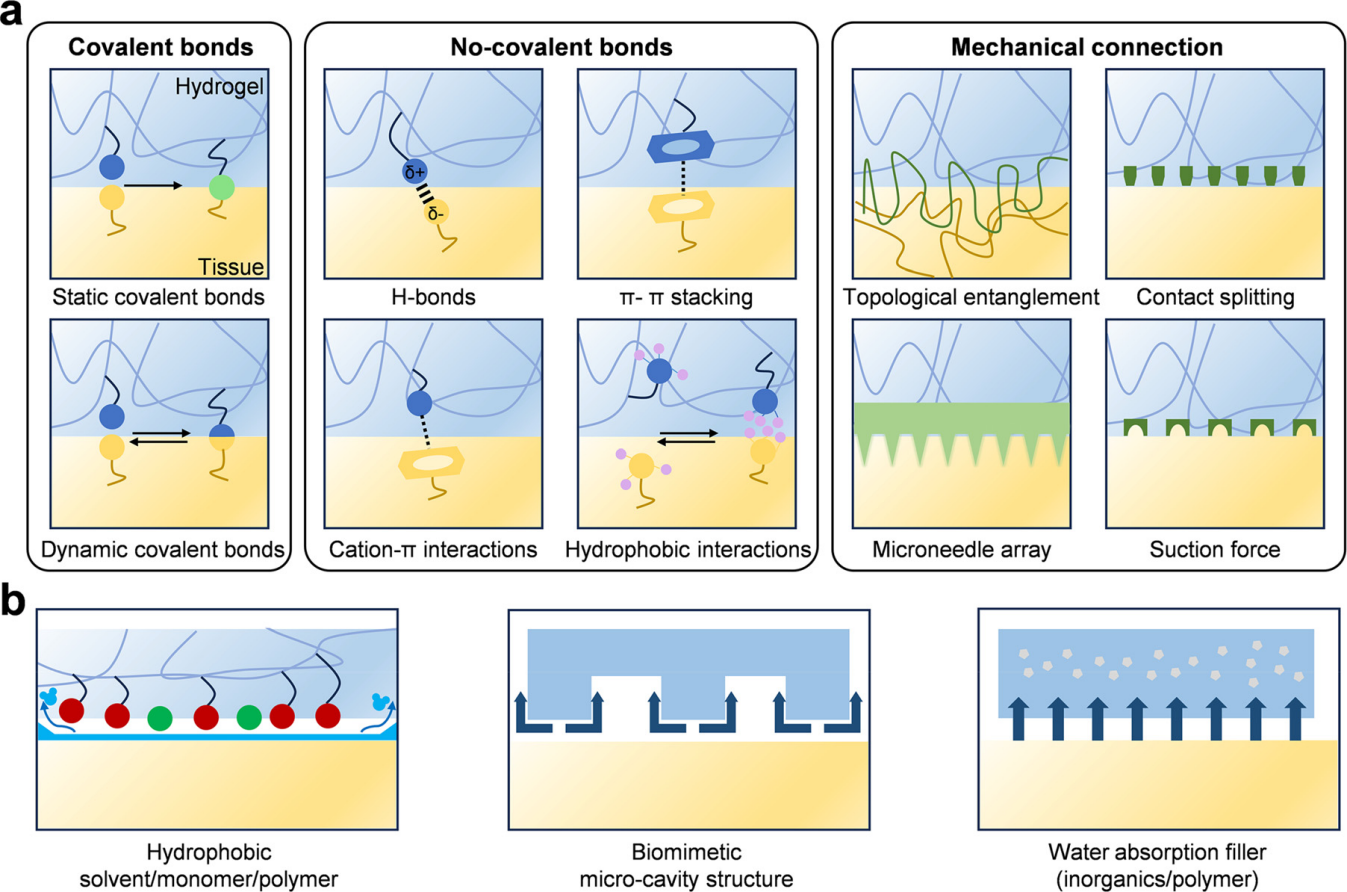
Interfacial adhesion mechanisms. (a) Physicochemical mechanisms for interfacial bonding and adhesion enhancement, including covalent/dynamic covalent chemistry, non-covalent interactions, and physical–mechanical interlocking strategies. Adapted with permission from Wu *et al.*, Chem. Rev. **123**(24), 14084–14118 (2023). Copyright 2023 American Chemical Society.^15^ (b) Adhesion strategies in wet environments involving hydration-layer repulsion and hydration-layer absorption. Adapted with permission from Fan *et al.*, Adv. Mater. **33**(44), 2102983 (2021). Copyright 2021 Wiley-VCH GmbH.^67^

Covalent bonds are the strongest and most stable interactions between the hydrogel network and the tissue matrix [Fig. 2(a)].^15^ They form between the functional groups of polymer chains (aldehydes, carboxylates, catechols, boronic acids, NHS esters) and the nucleophilic amino and thiol groups on tissue proteins.^41–43^ After formation, such bonds are usually irreversible unless specific chemicals or stimuli-responsive conditions are encountered to trigger them. Covalent coupling with the nucleophilic groups on the tissue surface (mainly lysine ε-amines and cysteine thiols) is a direct strategy to achieve durable adhesion, because a stable chemical bridge can be established between the adhesive matrix and the surface proteins. NHS-activated esters are often used in the chemical methods for *in situ* tissue bonding.^44^ The application of the precursor solution enables NHS esters to rapidly react with primary amines on the tissue surface under physiological to mild alkaline conditions, and then form stable amide bonds. This reaction is used to cross-link the adhesive matrix and anchor it to the tissue, and is the basis for the performance of commercial sealants such as DuraSeal™ and CoSeal™.^17^ However, NHS esters are prone to hydrolysis, and their effective reaction competes with water-mediated inactivation—strategies such as *in situ* generation and encapsulation are needed to maintain activity. Aldehyde groups generated by periodate oxidation of polysaccharides can easily react with tissue amines to form imine bonds. These dynamic bonds can achieve rapid underwater adhesion without external catalysts.^45,46^ Oxidized polysaccharides, such as oxidized dextran, alginate, and cellulose, can quickly seal bleeding tissues under physiological conditions. However, the Schiff base bond will hydrolyze in long-term aqueous solution, and the adhesion will disappear. Therefore, the designer combines the imine bond with secondary stable interactions, such as borate esters and hydrazone bonds. Covalent bonding-based adhesive strategies are widely applied in wound closure and surgical sealing, where strong and durable tissue fixation is required.^41^ Representative applications include aldehyde- or catechol-mediated cross-linking systems used for rapid hemostasis and long-term tissue integration in soft tissue repair.

Dynamic covalent bonds have the ability of stimuli-responsive reversibility and self-healing, and can construct intelligent adhesive interfaces. Boronic acid phenyl ester functionalized polymers can form reversible covalent boronate ester bonds with cis-diols on tissue glycoproteins and carbohydrates.^47–50^ This bonding is pH-dependent, is stronger at high pH values, can dissociate under acidic conditions, and is suitable for controlled peeling in acidic pathological environments and the stomach. Disulfide bond exchange is a mechanism. In the wound microenvironment, the redox conditions mediate this dynamic covalent mechanism. When there are thiol groups (–SH) on the hydrogel and tissue surface, disulfide bonds are formed under mild aerobic conditions. In a reductive wound environment, a high concentration of glutathione causes disulfide bonds to break, and the hydrogel dressing will degrade on demand and be removed non-invasively.^51^ Because it contains such dynamic modules, the hydrogel can maintain short-term stability and can also be controllably remodeled and removed. Dynamic covalent bonding-based adhesives have been extensively explored for applications involving dynamic or mechanically active tissues, owing to their reversible and self-healing characteristics. Such systems are particularly suitable for tissues undergoing repetitive deformation, such as skin and internal organs, where adaptable adhesion and stress dissipation are desirable.^51^

Inspired by mussel adhesive proteins, catechol chemistry has excellent wet adhesion performance relying on covalent- and coordination-based interactions.^52,53^ Catechol can be oxidized to quinones enzymatically, and the quinones undergo Schiff base condensation or Michael addition reactions with tissue nucleophiles. They can form strong metal–catechol coordination compounds (such as with Fe^3+^ and Ti^4+^) and participate in various hydrogen bonds and π interactions. The multivalent interactions enable the catechol-functionalized biopolymers to penetrate the hydrated layer and also come into contact with various substrates. The key point is that the oxidation state of catechol controls the binding mode and reversibility, which is a key design parameter for regulating adhesion and self-healing ability.

#### 2. Non-covalent interactions

Covalent bonds result in relatively high interfacial strength. Non-covalent bonds endow materials with flexibility, toughness, and self-healing properties. Individually, non-covalent bonds are relatively weak, but high density and fast binding/dissociation kinetics are crucial for physiological motion energy dissipation and dynamic reconstruction.^54–56^ In polysaccharide and protein hydrogels, hydrogen bonds and electrostatic interactions are common types of non-covalent bonds. Hydrogen bonds between polysaccharide hydroxyl/carboxyl groups and tissue protein amide groups can stabilize the interfacial network and enhance adhesion. These interactions are easily affected by the surrounding water environment, so increasing the density of polar binding sites and combining hydrogen bonds with other interaction types significantly enhances wet adhesion. A single hydrogen bond is relatively weak, but collective hydrogen bonds can act as sacrificial bonds, which break and re-form when stressed, thereby dissipating energy and preventing catastrophic failure at the interface. There is an electrostatic attraction between polymer segments with opposite charges and the tissue surface, which is a second fast and water-resistant anchoring mechanism. For example, electrostatic interactions will occur between positively charged CS and negatively charged cell membranes and extracellular matrix components. Excessive charge density may damage biocompatibility and also cause non-specific protein adsorption, which needs to be optimized properly.

In a humid situation, the hydrophobic interaction and the effect of interface water repulsion become highly effective. Introducing hydrophobic areas and oil phases can drive away the water layer, making the adsorption groups closely contact with the tissue surface.^57,58^ Aromatic-aromatic π–π stacking and cation–π interaction stabilize the interface relying on aromatic and charged residues, and can also form a synergistic multivalent network with hydrogen bonds and metal coordination. The key is that these non-covalent interactions are reversible, enabling the material to achieve self-healing and fatigue resistance. Weak bonds continuously dissociate and then re-form under mechanical stress. Non-covalent interaction-based adhesion, including hydrogen bonding, electrostatic interactions, and hydrophobic interactions, has been commonly employed in temporary wound dressings and bioadhesive patches.^55^ These interactions enable reversible tissue adhesion and minimize tissue damage during removal, making them attractive for short-term or repeat-use applications.

#### 3. Physical and mechanical interlocking

In addition to intermolecular interactions, mesoscopic and microscopic mechanical interlocking enhances adhesion by increasing the contact area and constructing energy dissipation paths that impede crack propagation.^59,60^ Topological entanglements occur when polymer chains diffuse to the surface of porous tissue and the second polymer network. During gelation and ionic cross-linking, interpenetrating chains form mechanical interlocking regions that resist separation.^61^ This strategy is particularly effective for porous/rough tissues and can also be enhanced by applying a stitching polymer layer (such as high-molecular-weight polyacrylic acid and CS), which forms hydrogen bonds and secondary interactions with the surrounding matrix. The key limitation is that entanglements require sufficient diffusion time, which is less effective for dense/low-permeability tissues, prompting the use of methods such as ultrasound activation and solvent-assisted infiltration to accelerate wetting.

Engineered microneedles, barbs, and micropillar arrays mechanically interlock with tissue microstructures, thus providing another case of physical adhesion.^62,63^ Inspired by biological systems such as plant hooks and insect tarsi, these structural adhesives have the characteristics of high peel and shear strength. For example, the tip of a swellable microneedle swells after absorbing liquid, has an anchoring effect, and also increases the pull-out resistance. Nanoparticles such as silica adsorb polymer chains and form partially mediated entangled networks, acting as physical bridges between hydrogels and tissues.^64^ The hydrogel, inspired by the octopus suckers, relies on geometric deformation and negative pressure to produce a case of reversible wet adhesion.^65^ These structures can block interface liquids and fit irregular, water-containing surfaces. They can improve interface strength and achieve reversible bonding, but their biomedical applications need to consider complex manufacturing and surface topography sensitivity. These systems are especially relevant for hemostatic applications and internal organ repair, where rapid displacement of interfacial water is critical for effective tissue bonding.

### B. Adhesion strategies in wet environments

In practical biomedical scenarios, the presence of blood and physiological body fluids poses a significant challenge to effective tissue adhesion. Interfacial water layers and hydrated biomolecules can prevent intimate contact between the adhesive material and tissue surface, thereby reducing bonding efficiency. In addition, proteins, lipids, and cellular components in blood may competitively adsorb onto tissue interfaces, shielding reactive functional groups and interfering with covalent or non-covalent interactions. These factors collectively compromise adhesion strength, durability, and reliability, particularly in dynamic and bleeding environments.^66^ To solve this problem, researchers proposed two complementary design strategies for high-performance bioadhesives [Fig. 2(b)]: (i) exclusion method, removing and replacing interfacial water for direct contact; (ii) absorption method, capturing and stabilizing interfacial water in the adhesive network.^67^ Such approaches are especially critical for hemostatic applications and internal organ repair, where rapid and stable adhesion under physiological conditions is required.

The exclusion of the hydration layer enables the tissue to achieve adhesive contact by actively removing the interfacial liquid.^68,69^ This exclusion can be achieved at multiple length scales. At the molecular level, hydrophobic groups expel water through hydrophobic interactions, and it can also be achieved by adding hydrophilic monomers and solvents.^70,71^ At a larger scale, hydrophobic microstructures are designed to repel the water layer.^72^ The hydrogel surface has a thin hydrophobic coating, and a low contact angle helps to displace the interfacial water under mild pressure. The coating makes the uncross-linked chains have strong fluidity, and various forces contribute to the combination of interfaces.^70^ Meanwhile, hydrophobic monomers with long alkyl and aromatic groups can prevent the entry of water.^73,74^ For example, Fe^3+^ causes the OMA chains in SDS micelles to aggregate, forming dynamic hydrophobic connections to exclude water from the interface.^73^ Similarly, the aromatic groups reduce the local dielectric constant, enhance π–π and other effects, and strengthen the contact between molecules.^74^ The biomimetic microcavity structure originates from various organisms, such as octopus,^75^ clingfish,^76^ tree frog,^77^ and gecko.^78^ These biological systems provide inspiration for designing microstructured interfaces in biobased adhesive materials, rather than serving as direct structural replicas. At the macroscopic level, such microcavity architectures facilitate water removal through capillary and siphon-like drainage pathways. The central contact region formed at the interface generates negative pressure, creating localized dry contact points that enable strong and reversible underwater adhesion, as well as effective energy dissipation. Importantly, these principles have been translated into biobased adhesive hydrogel designs to improve adhesion performance under wet and dynamic physiological conditions. Such designs are particularly relevant for mechanically active organs, including the beating heart and contracting lung, where repeated deformation and interfacial fluid presence pose significant challenges. For example, Wang *et al.* developed cup-shaped microcavities that undergo elastic deformation when compressed, which can expel trapped water and increase the contact area.^75^ Similarly, hydrogels with hexagonal patterns have interconnected grooves, like the sucker pads of sticky fish and frogs, which help in rapid water drainage, limit cracks, and enhance fatigue resistance.^76,77^ In this review, such biomimetic microstructural strategies are discussed primarily as design concepts informing biobased adhesive systems, rather than as detailed biological models, with emphasis placed on their functional relevance to wet-tissue adhesion.

There is a clever way to deal with the water-hydration layer: instead of removing the interfacial water, let the hydrogel network absorb and fix it, and transform the interfacial liquid film into the adhesive part.^79^ This is achieved by forming an interpenetrating network. The network and hydrophilicity make water stay and ensure the contact between functional groups and tissue proteins. Polymers such as poly(vinylpyrrolidone) (PVP),^80^ poly(ethyleneimine) (PEI),^79^ and polyacrylamide (PAAm)^81^ are often added to enhance the hydrophilicity. In order to improve the effect, it is made into a dry form (such as a dry adhesive and powder), which absorbs the water-hydration layer when in contact and quickly forms a hydrogel *in situ*. For example, a dried double-sided tap composed of gelatin blended with NHS-grafted poly(acrylic acid) (PAAc-NHS) can dry wet tissues under a mild pressure of about 1 kPa, and immediately form interfacial hydrogen bonds and electrostatic bonds through carboxylate–amine interactions.^53^ Similarly, self-gel PEI/PAAc powder absorbs interfacial water within 2 s and forms adhesive hydrogel through polymer diffusion and physical association.^82^ Incorporating quaternized chitosan (QCS) can enhance blood absorption and the concentration of clotting factors and can *in situ* gel, stop bleeding, and achieve mechanical sealing in a humid environment.^66^ In essence, this method internalizes interfacial water, and the hydrogel achieves seamless integration with tissues without replacing bound water, and is suitable for fragile tissues that cannot withstand drying and high pressure.

In summary, the strong wet adhesion of biobased hydrogels is rarely achieved by a single mechanism. Instead, advanced bioadhesives combine covalent anchoring (for long-term fixation), reversible non-covalent interactions (for toughness and self-repair), mesoscopic mechanical interlocking (for fracture resistance), and practical interfacial water management strategies (for maintaining stable contact in humidity). Despite the significant progress achieved by advanced adhesive strategies, several inherent limitations must be carefully considered for practical and clinical applications. For example, aldehyde-based chemistries, while enabling rapid covalent bonding with tissue amines, may raise concerns regarding cytotoxicity and inflammatory responses when residual reactive groups remain. Similarly, catechol-based adhesives, inspired by mussel adhesion, can undergo oxidation under physiological conditions, potentially leading to reduced adhesion stability and unpredictable biological effects. In addition, mechanical mismatch between adhesive materials and soft tissues can result in stress concentration, delamination, or impaired tissue healing, particularly in dynamic organs. Long-term stability of the adhesive interface remains another challenge, as swelling, fatigue, or hydrolytic degradation may compromise adhesion performance over time. Furthermore, uncontrolled or excessively rapid degradation may lead to premature loss of mechanical support, whereas overly slow degradation may hinder tissue regeneration or clearance. Addressing these limitations requires integrated design strategies that balance adhesion strength, biocompatibility, mechanical compliance, and degradation behavior.

## III. EVALUATION METHODS FOR BIOBASED ADHESIVE HYDROGELS

Before clinical application, there needs to be a comprehensive and standardized evaluation framework to evaluate the mechanical properties, hemostatic properties, biocompatibility, and degradation behavior of biobased adhesive hydrogels. These materials function in complex biological environments and need to find an exact balance among stickiness, elasticity, and biodegradability. Strict *in vitro* and *in vivo* characterizations provide key insights into performance, stability, and safety. With the emergence of more complex material designs and functional requirements, evaluation methods are continuously evolving to guide the optimization of hydrogels and facilitate clinical translation.

### A. Mechanical and adhesive performance testing

**FIG. 3.**
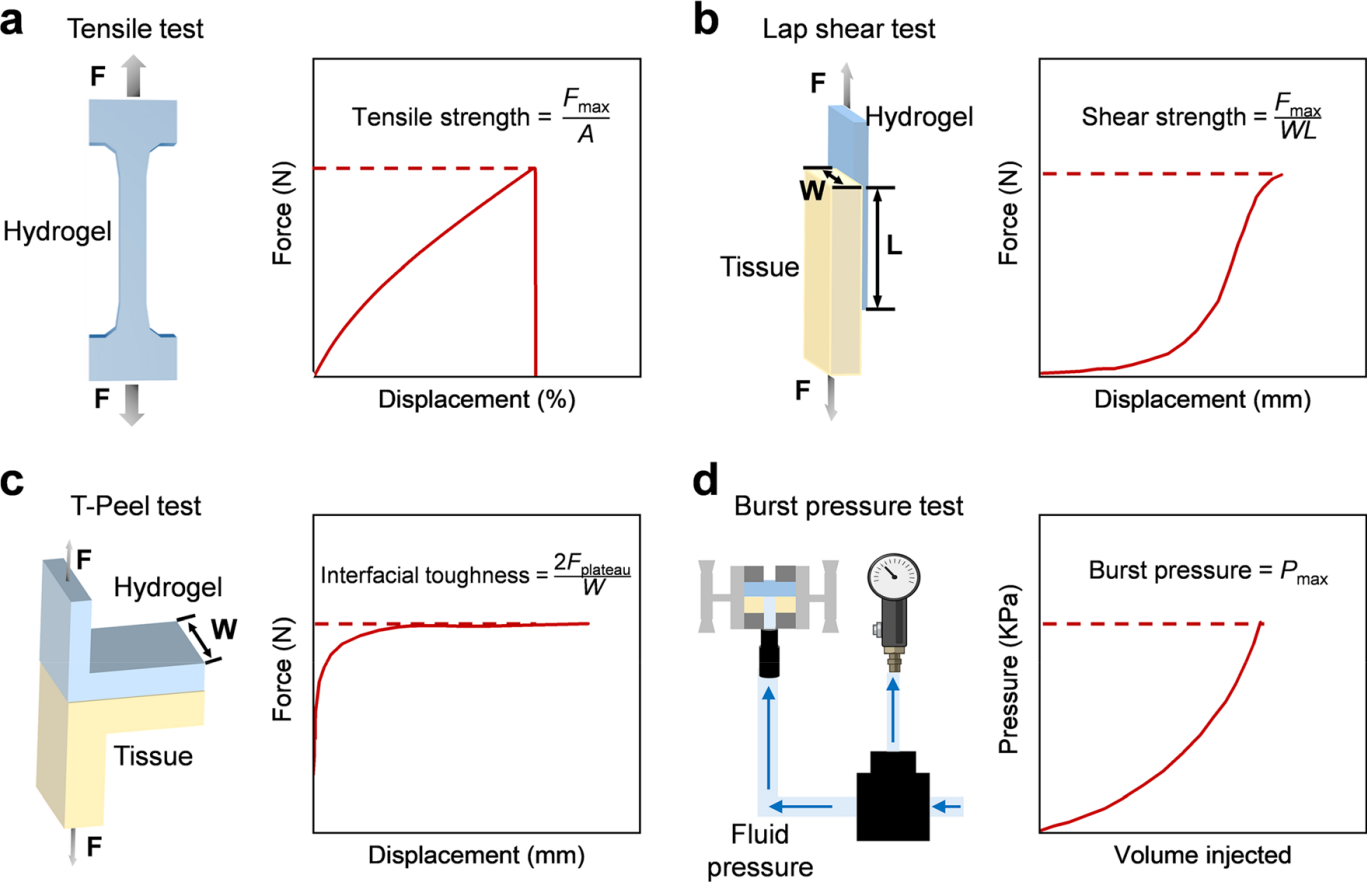
Experimental setup for evaluating the mechanical and adhesive properties of hydrogels. (a) Intrinsic stress–strain is determined by tensile testing (ISO 37).^83^ (b) Shear strength is determined using lap shear testing (ASTM F2255).^84^ (c) Interface toughness is evaluated using T-type peel testing (ASTM F2256).^85^ (d) Burst pressure of the adhesive is tested by burst pressure test (ASTM F2392).^86^

Mechanical properties will have an impact on the durability and stress resistance of biobased adhesive hydrogels. Standardized mechanical tests can quantify relevant parameters. The overall elasticity and other situations are evaluated through tensile stress–strain characteristics (ISO 37), and indicators such as ultimate tensile strength and elongation are given [Fig. 3(a)].^83^ These parameters can reflect the deformation durability of the material during tissue movement, which is important for load applications such as cartilage. Double-network and nano-composite hydrogels usually have extremely high tensile properties, with an elongation at break exceeding 500% and toughness close to that of soft tissue.

Adhesive performance is measured by lap shear testing (ASTM F2255). In this test, two base materials are bonded with hydrogel, and shear is performed at a constant speed until failure [Fig. 3(b)].^84^ The maximum shear stress at this time is the adhesive strength. The adhesive strength of high-performance biobased hydrogels is between 20 and 100 kPa, and the specific value depends on the chemical composition and test conditions. T-peel testing (ASTM F2256) is used to measure the interfacial peel strength [Fig. 3(c)].^85^ This test simulates the force of separation and deformation. A situation with high peel strength and no brittle fracture indicates that energy is dissipated through reversible interactions, which is very important for skin and soft tissue applications. The burst pressure test (ASTM F2392) is very important for vascular and visceral sealants [Fig. 3(d)].^86^ The hydrogel is placed at the defect of the elastic membrane, and the pressure is gradually increased until leakage occurs. When the burst pressure of adhesive hydrogel exceeds 50–120 kPa, it can seal arteries and high-pressure wounds. Advanced research includes fatigue and cyclic load tests to evaluate long-term durability and elastic recovery, which is related to continuously moving tissues such as the heart and blood vessels. Combining these mechanical tests can comprehensively understand the behavior of hydrogels under different loading conditions and help design strategies for specific tissue environments.

### B. Hemostatic performance and *in vivo* wound models

The evaluation of the hemostatic effect of colloidal hydrogel is often combined with *in vitro* coagulation analysis and animal hemorrhage models to explore rapid blood coagulation and wound closure. *In vitro* measurement includes the activated partial thromboplastin time, prothrombin time, and blood loss.^87,88^ Compared with the control group, shorter clotting time and less blood loss indicate a stronger hemostatic effect. The adhesion and activation of platelets can be analyzed by SEM and flow cytometry to explore the enhanced blood coagulation mechanism.

The animal hemorrhage models for simulating surgery used in *in vivo* evaluation commonly include situations such as liver and spleen incision, and femoral artery puncture in rats and pigs, with different degrees of hemorrhage severity and complexity.^89,90^ These models can quantitatively evaluate hemostasis time, blood loss, rupture, and leakage under physiological pressure. The models of liver and artery injury are suitable for evaluating the effect of immediate hemostasis and sealant occlusion under high flow. After application, drainage and leakage tests are carried out to further evaluate watertightness, which is the key to blood vessel and gastrointestinal repair. These evaluations provide strong evidence for the effectiveness and safety of biobased adhesive hydrogels as hemostatic agents.

### C. Biological evaluation

Clinical transformation of biobased adhesive hydrogels requires inherent biocompatibility and safety. Biocompatibility often relies on *in vitro* cell compatibility tests and *in vivo* tissue reaction evaluations. Cytotoxicity is commonly detected by MTT, CCK-8, and live/dead staining methods.^91,92^ The cell proliferation and viability are evaluated through hydrogel extract exposure and direct contact. Commonly used mammalian cell lines include fibroblasts (L929), keratinocytes, and epithelial cells. According to the ISO 10993-5 standard, hydrogels with cell viability greater than 90% are non-cytotoxic and can be used for further biological research.^93^ Hemolysis test (ASTM F756) evaluates red blood cell damage by measuring hemoglobin release; a hemolysis rate below 5% is clinically acceptable.^94^

Immune and inflammatory responses belong to key biological indicators. The expression of pro-inflammatory cytokines, such as TNF-α, IL-6, and macrophage polarization, can show the effect of immune regulation.^95^ Hydrogels containing natural polysaccharides and phenolic groups usually promote macrophage polarization and relieve inflammation. Histological analyses, such as hematoxylin and eosin (H&E) and Masson's trichrome staining, can observe tissue integration, collagen deposition, and inflammation cell infiltration at the implantation site.^96^ Immunohistochemical analyses of markers such as CD31, VEGF, and α-smooth muscle actin are used to clarify angiogenesis and tissue remodeling, providing evidence for the mechanism of regeneration potential. Based on these evaluation results, the short-term and long-term safety and regeneration compatibility of the biobased hydrogel are determined.

### D. Degradation and stability assessment

Biobased hydrogels have biodegradable properties, can temporarily assist tissue regeneration, and are then absorbed without causing adverse effects.^44^ Degradation is usually evaluated under hydrolysis, enzymatic hydrolysis, and oxidation conditions to simulate physiological and pathological environments. The adopted scheme needs to be compatible with the chemical characteristics of the polymer.

During the hydrolysis degradation test, the sample is immersed in PBS buffer with a pH of 7.4 at 37 °C. The mass loss, swelling degree, and mechanical properties are measured over time.^63^ It is studied that the ester bonds, amide bonds, and imine bonds in the polymer main chain exhibit stability. Enzymatic degradation can simulate the *in vivo* environment, and natural polymers are easily acted on by specific enzymes, such as lysozyme (for CS), hyaluronidase (for HA), and collagenase (for gelatin and collagen).^97^ The degradation rate can be quantitatively analyzed by residual mass, gel permeation chromatography, and spectrophotometric analysis of degradation products. Using reactive oxygen species (ROS) to simulate substances like H_2_O_2_ for oxidative degradation evaluation, these simulated substances can reflect the inflammatory microenvironment. This is relatively important for catechol cross-linked hydrogels, which are easily decomposed by ROS-mediated oxidation. Controlled degradation provides sufficient mechanical support during tissue repair and prevents the formation of chronic inflammation and fibrotic capsules after regeneration. With the passage of time, the degradation kinetics is optimized by using SEM and FTIR to coordinate with the tissue-specific regeneration rate: skin and mucosa need rapid degradation, while bone and cartilage need slow degradation. Figuring out the interaction between degradation, adhesion, and biological response is the key to developing safe and high-performance bioadhesive hydrogels.

Although standardized mechanical, hemostatic, and biological tests provide useful benchmarks for evaluating adhesive performance, their relevance to complex *in vivo* tissue environments remains limited. Most *in vitro* adhesion tests are conducted on simplified, static tissue substrates under controlled conditions, which do not fully capture the heterogeneity, hydration, vascularization, and dynamic motion of living tissues. As a result, adhesion strengths measured *in vitro* may overestimate performance in physiological settings, particularly for wet or bleeding tissues. Furthermore, direct comparison of adhesion data across different studies is often challenging due to inconsistent testing conditions. Variations in tissue type and source, surface preparation, sample geometry, loading mode (lap shear, tensile, or peel), strain rate, and environmental conditions can lead to significant discrepancies in reported values. The lack of unified testing standards complicates objective performance benchmarking and may hinder rational material selection and translation. Future efforts should prioritize the development of more physiologically relevant testing models and standardized evaluation protocols that better reflect clinical scenarios, enabling more meaningful comparison and accelerating translational progress.

## IV. NATURAL POLYMERS FOR BIOBASED ADHESIVE HYDROGELS

Biobased adhesive hydrogels are derived from natural polymers. Its molecular structure balances biocompatibility, degradability, and adjustability. Polymers, mostly polysaccharides and proteins, have reactive groups for chemical modification and cross-linking. The hydration behavior, charge distribution, and mechanical properties are similar to the extracellular matrix.^98–100^ Here, the main categories of natural macromolecules in adhesive hydrogels will be presented, as well as how chemical modification and composition strategies achieve functional design for biomedical applications. For clarity, Table II compares the major biobased adhesive platforms discussed in Sec. IV, highlighting their key pros, limitations, and the wound/tissue conditions for which each is most suitable.

**TABLE II.** Comparison of representative biobased adhesive hydrogels and their suitable application conditions.

Type	Materials	Advantages	Limitations	Applications	Ref.
Polysaccharide	CS	Cationic hemostasis; antibacterial effect; easy functionalization for wet adhesion	Physiological insolubility; weak mechanics; needs blending and cross-linking	Wet bleeding wounds; infection-prone sites; rapid sealing and hemostasis required	101, 102
	Cellulose	High-strength fibrous scaffold; water retention; supports composite reinforcement	Limited intrinsic adhesion; requires oxidation or blending for tissue bonding	Wound dressings; structural support layers; composites needing durability and moisture	103, 104
	Alginate	Fast Ca^2+^ gelation; injectable conformal filling; tunable with oxidation	Ion-exchange softening; low toughness; needs secondary network reinforcement	Irregular defects; *in situ* hemostasis; rapid gelation under wet conditions	105, 106
	HA	ECM-mimetic hydration; supports cell migration; tunable gelation and degradation	Native HA weak; rapid enzymatic degradation; requires cross-linking modification	Moist wounds or mucosa; regeneration cues; gentle chemistry with tunable residence	107, 108
Protein	Gelatin	Cell-adhesive motifs; photo-tunable stiffness; supports regenerative microenvironment formation	Unmodified gelatin dissolves; photoinitiator and light access constraints	Low-to-moderate stress repair; *in situ* sealing; tissue engineering scaffolds	109
	SF	High strength; slow degradation; maintains integrity under cyclic deformation	Adhesion insufficient; needs functionalization or blending to bind tissues	Dynamic tissues, cardiac or vascular; long residence and mechanical retention	110
	Fibrin sealants	Clinically used; rapid gelation; biocompatibility for hemostasis	Limited strength and wet adhesion; weak for high-pressure sealing	Adjunct hemostasis; low-pressure sites; fast intraoperative handling needed	111
Multifunctional and stimuli-responsive	Temperature-responsive	Mild trigger; injectable sol–gel transition; supports on-demand payload release	Narrow transition window; thermal stability varies with formulation and loading	Minimally invasive injection; in situ gelation at body temperature sites	112, 113
	pH-responsive	Pathology-linked response; conditional swelling; enables targeted release and adhesion	Buffering by fluids; pH gradients small; reduced response robustness	Inflamed or tumor microenvironments; localized delivery; pH-guided therapy	114, 115
	Light-responsive	High spatiotemporal control; on-demand curing; precise release modulation	Limited penetration depth; photothermal overheating risk; requires external light	Accessible tissues; localized sealing; imaging-guided or minimally invasive procedures	116
	Magnetic-responsive	Remote actuation; deep-tissue potential; controlled positioning and release	Requires magnetic particles; field equipment; heating and safety constraints	Deep tissues; targeted delivery; non-invasive control under magnetic fields	117, 118
	Ultrasound-responsive	Deep penetration; non-invasive trigger; compatible with imaging-guided release	Parameter-sensitive; repeated stimulation may weaken networks and adhesion	Deep sites; ultrasound-guided therapy; pulsatile release on demand	119

### A. Polysaccharide-based adhesive hydrogels

**FIG. 4.**
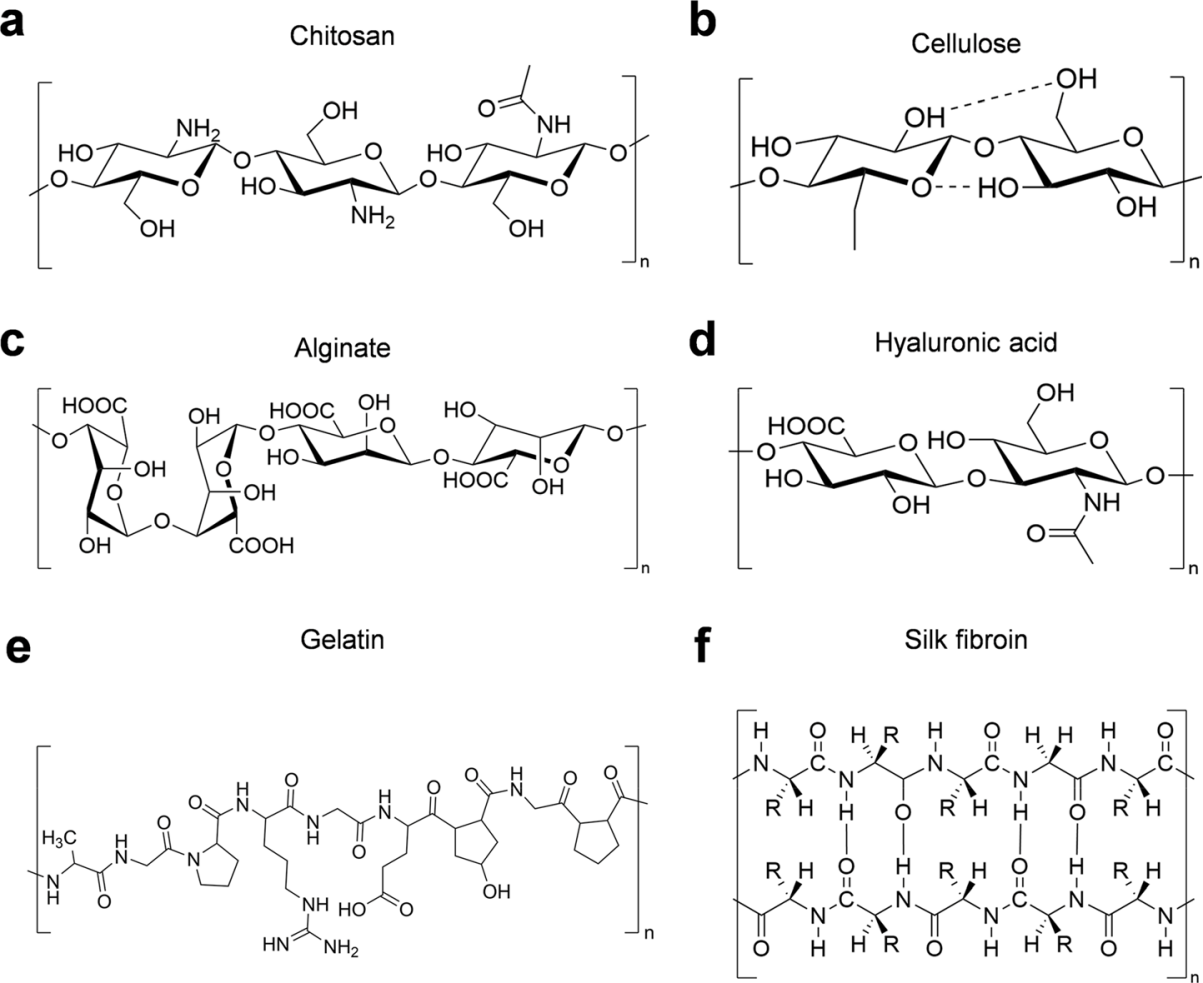
Chemical structures of biobased materials used for adhesive hydrogel fabrication. Polysaccharides include (a) CS, (b) cellulose, (c) HA, and (d) alginate; proteins include (e) gelatin and (f) silk fibroin.

In Fig. 4(a), CS is a deacetylated derivative of chitin that has been widely studied for bioadhesive hydrogels. The primary amine groups carry cationic charges, which can have electrostatic interactions with negatively charged cell membranes and extracellular glycoproteins.^101,120^ This positive charge activates platelets and red blood cells to enhance interfacial adhesion and promote hemostasis. Therefore, CS-based adhesives are widely explored as rapid surgical sealants in forms such as coatings, injectable hydrogels, and electrospun fibers.^121,122^ However, natural CS is only soluble in acidic environments and has limited mechanical strength. Common chemical modifications, such as quaternization, carboxylation, and catechol functionalization, are used to overcome the limitations. QCS like N,N,N-trimethyl CS has permanent positive charges and does not change with pH, remains in a sol state under physiological conditions, and can also disrupt microbial membranes to enhance antibacterial activity. Catechol-functionalized CS with a dopamine moiety mimics mussel foot proteins and produces strong wet adhesion through covalent and non-covalent interactions.^102^

Cellulose and its derivatives, such as carboxymethyl cellulose (CMC) and hydroxyethyl cellulose (HEC), are abundant and renewable biopolymers with high crystallinity and good mechanical strength [Fig. 4(b)].^103,123^ Within adhesive hydrogels, cellulose is either a structural framework and a water-retaining matrix. Oxidized cellulose and dialdehyde cellulose have reactive aldehyde groups, which can form covalent Schiff base bonds with amine-rich proteins. Bacterial cellulose produced by Acetobacter xylinum has a nanoscale fiber network and excellent tensile strength, porosity, and water retention.^104,124^ The purity and three-dimensional microfiber structure make bacterial cellulose an ideal scaffold for tissue regeneration and can enhance the skeleton in hydrogel composites. Bacterial cellulose combined with CS, gelatin, and hydroxyapatite can enhance mechanical integrity and provide a porous, biocompatible structure, which is beneficial to the diffusion of nutrients and the infiltration of cells. In addition, modifying the bacterial cellulose surface with catechol and silyl groups can further improve its adhesion to soft tissues and the compatibility of the interface.

Alginate in brown algae is a block copolymer of β-D-mannuronic acid (M) and α-L-guluronic acid (G) residues [Fig. 4(c)].^105^ It can form ionically cross-linked hydrogels through divalent cations such as Ca^2+^. The egg-box coordination between guluronic acid residues and cations contributes to immediate gelation and can adjust the hardness. This rapid ionic cross-linking can immediately solidify and stop bleeding at the bleeding site, making alginate the top polysaccharide substrate for injectable sealants. Partially oxidized alginate with aldehyde groups can react with histamine and amino-containing polymers to enhance covalent adhesion.^106^ Oxidized alginate cross-linked by Ca^2+^ ions has synergistic hemostatic and adhesive properties. Ionic bonds endow cohesive strength, and imine bonds maintain interface fixation. In order to enhance toughness and biological functionality, alginate is often blended with gelatin and CS to form double networks and polyelectrolyte complexes with rapid gelation and intact structure. The biocompatibility, easy functionalization, and controllable gelation kinetics of alginate make it a multifunctional platform for clinical wound dressings and surgical sealants.

HA is a linear glycosaminoglycan composed of D-glucuronic acid and N-acetyl-D-glucosamine [Fig. 4(d)].^107^ It is a key extracellular matrix component for maintaining water and cell signal transduction. HA has high hydrophilicity and good biocompatibility, and is suitable as an adhesive for wound healing and tissue repair that must maintain a moist environment. Natural HA can promote cell proliferation, angiogenesis, and inflammation regulation, but it has poor mechanical strength and fast degradation rate, so cross-linking modification is required.^108,125^ Methacrylated HA (HAMA) has a photocrosslinkable vinyl group and can *in situ* gel under ultraviolet/visible light. The hardness and degradation rate can be controlled by adjusting the substitution degree and irradiation parameters. By oxidizing with periodate to obtain aldehyde-modified HA, it can form Schiff base bonds with amino polymers and tissue proteins, and then prepare hydrogels with rapid adhesion and controllable degradation. Beyond structural support, HA interacts with cell surface receptors such as CD44, promoting cell migration, proliferation, and blood vessel formation.^126^ Therefore, HA-based adhesive hydrogels usually not only have the function of mechanical sealing but can also actively guide tissue regeneration.

### B. Protein-based adhesive hydrogels

As shown in Fig. 4(e), gelatin is a derivative of partially denatured collagen, which has numerous carboxyl groups and amino groups, as well as a bioactive RGD sequence that can help cell adhesion, migration, and remodeling.^109^ Since it is derived from the extracellular matrix, gelatin has inherent biocompatibility and activity. However, unmodified gelatin is easily soluble in water and has weak mechanical properties, so chemical cross-linking is the key to forming stable hydrogels. Gelatin methacryloyl (GelMA), a commonly used derivative, can form hydrogels with adjustable hardness and degradation rate through photo-induced cross-linking.^127^ When used together with polysaccharides containing aldehyde groups, GelMA forms a covalent-ionic hybrid network for strong wet-tissue adhesion through amide bonds and hydrogen bonds. Natural collagen has a triple-helix structure, remains, and acts as an enhancing matrix, providing mechanical stability and biochemical signals for angiogenesis and extracellular matrix deposition. In composite adhesives, collagen provides load-bearing for the fibrous form, and functional polysaccharides and catechol moieties provide adhesion for the interface.

The silk fibroin in the silkworm cocoon has the characteristics of high mechanical strength, slow degradation, and good biocompatibility. The β-sheet nanocrystalline domains enable it to stretch like natural tendons and become a useful additive for strengthening hydrogels [Fig. 4(f)].^127^ After functional modifications such as methylation and tyrosine oxidation, and blending with polysaccharides, a cross-linked network with better elasticity and adhesion can be formed.^110^ Silk fibroin is composed of amino acids including glycine, alanine, and serine, which help connect with carbonyl and aldehyde groups and achieve tissue adhesion. In adhesives, silk fibroin is a deformable scaffold, and the added functional groups and second polymers help to enhance the interface bonding force and biological activity. This type of system is used as a cardiac patch and vascular sealant and can adhere persistently under cyclic deformation. The characteristics of silk fibroin are slow degradation and high tensile strength, which can improve the integrity of the hybrid hydrogel structure.

Thrombin polymerizes fibrinogen into fibrin, which has the properties of hemostasis and cell adhesion and is a biological adhesive in the early clinical stage.^128,129^ Commercial fibrin sealants such as Tisseel^®^ and Evicel^®^ are often used in surgery for hemostasis and wound closure because of their good biocompatibility and ability to gel quickly.^111^ However, natural fibrin gels have poor mechanical strength and adhesion effects in a high humidity environment, so their application in high-pressure wounds is limited. Recent research focuses on hybridizing fibrin with other natural polymers and nanoparticles to overcome limitations. For example, adding oxidized polysaccharides and enzyme-modified hydrogels to enhance adhesion strength, and embedding carbon nanostructures to enhance mechanical properties.^130^ Fibrin-based adhesives have clinical relevance, and its improved structural stability and long-term stability remain important research directions.

### C. Multifunctional and stimuli-responsive biobased adhesive hydrogels

Biobased adhesive hydrogels are transitioning from single-function tissue sealants. By integrating stimuli-responsive characteristics into the framework of natural-source polymers, researchers have developed a new generation of bioadhesive materials, which can sense and interact dynamically with biological systems.^131,132^ These hydrogels can adjust adhesion strength, prevent infection, and control degradation under external and internal stimuli, meeting the needs of precision medicine. This part summarizes the latest research progress of temperature-, pH-, light-, magnetic-, and ultrasound-responsive biobased hydrogels.

**FIG. 5.**
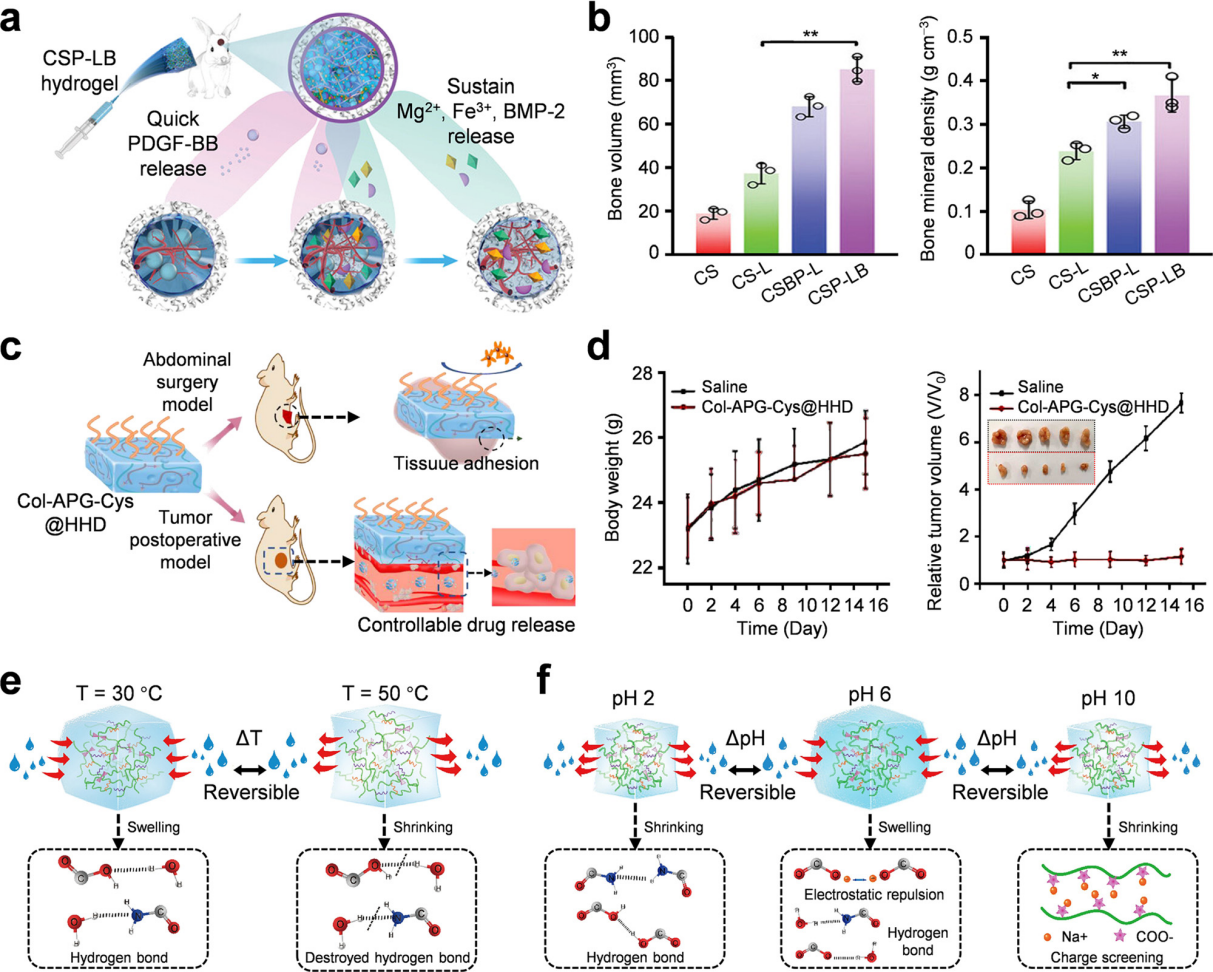
Stimuli-responsive biobased adhesive hydrogel. (a) Schematic diagram of temperature-responsive hydrogel for bone regeneration, which can rapidly release growth factors. (b) Micro-CT analysis of new tissue bone volume and mineral density. (a) and (b) Adapted with permission from Lv *et al.*, Adv. Mater. **35**(5), 2206545 (2023). Copyright 2022 Wiley-VCH GmbH.^134^ (c) Schematic diagram of Col-APG-Cys@HHD double-layer hydrogel for anti-adhesion and anti-tumor recurrence. (d) Mice were treated with normal saline and Col-APG-Cys@HHD, and body weight changes and tumor growth curves were observed. (c) and (d) Adapted with permission from Zhou *et al.*, Acta Biomater. **158**, 228–238 (2023). Copyright 2022 Elsevier.^136^ Semi-fiber-based hydrogels with (e) temperature response and (f) pH response mechanisms. (e) and (f) Adapted with permission from Chen *et al.*, Carbohydr. Polym. **247**, 116717 (2020). Copyright 2020 Elsevier.^137^

Temperature-responsive hydrogels undergo reversible sol-gel phase transitions in the presence of the lower critical solution temperature (LCST).^112^ Below the LCST, they are in a liquid state, and above the LCST, they form a gel state. This temperature-responsive hydrogel does not require the use of toxic initiators, and the operation process is relatively mild and biocompatible. It can be administered by injection, and *in situ* gelled at sites where the local temperature is higher than the LCST. Therefore, this material has been extensively applied in drug sustained release and regenerative medicine.^113,133^ Lv *et al.* developed a temperature-responsive hydrogel that combined the functionalized MgFe-layered double hydroxide (LDH) nanosheets [Fig. 5(a)].^134^ Incorporation of MgFe-LDH reduces the gel time from 300 s to 146 s and also reduces the LCST to 32.7 °C, enabling rapid sol-gel transition at body temperature and enhancing *in vivo* stability. This temperature-responsive hydrogel also assisted angiogenesis and osteogenesis for bone defect repair [Fig. 5(b)], offering a promising minimally invasive clinical strategy for bone regeneration.

The change of local pH is a key biochemical signal in pathological environments such as tumors, inflamed tissues, and intracellular components.^114^ The physical and chemical properties of pH-responsive hydrogels will change under acidic or alkaline conditions, and they have important application value in targeted controlled-release drug delivery.^115,135^ Zhou *et al.* cross-linked collagen and recombinant albumin nanoparticles (HHD NPs) with aldehyde-functionalized polyethylene glycol (APG6K) and attached zwitterionic cysteine (Cys) on the surface to prepare pH-sensitive Col-APG-Cys@HHD hydrogels [Fig. 5(c)].^136^ Surfaces modified with Cys can prevent postoperative peritoneal adhesion. The unmodified surface adheres to the acidic tumor resection site, realizing local sustained drug release. *In vivo* studies confirm that Col-APG-Cys@HHD can effectively prevent postoperative tumor recurrence and peritoneal adhesion [Fig. 5(d)], creating a transformable platform for deep abdominal tumor treatment.

Stimuli-responsive hydrogels are classified into single, dual, and multi-responsive types according to the stimulus type. Multi-responsive hydrogels often combine natural polymers with environmentally sensitive molecules to form adaptive materials. Chen *et al.* extracted hemicellulose from wastewater of paper mills, cross-linked it with acrylamide and acrylic acid, and prepared pH/temperature dual-responsive hydrogels.^137^ The carboxyl group endows the hydrogel with protonation-swelling properties and can be sensitively responsive to the pH value of the environment [Fig. 5(e)]. This hydrogel has a temperature-related swelling ratio because there are temperature-related hydrogen bond changes between the hydrophilic groups and water molecules [Fig. 5(f)], which also means potential applications in smart packaging and biomedicine.

**FIG. 6.**
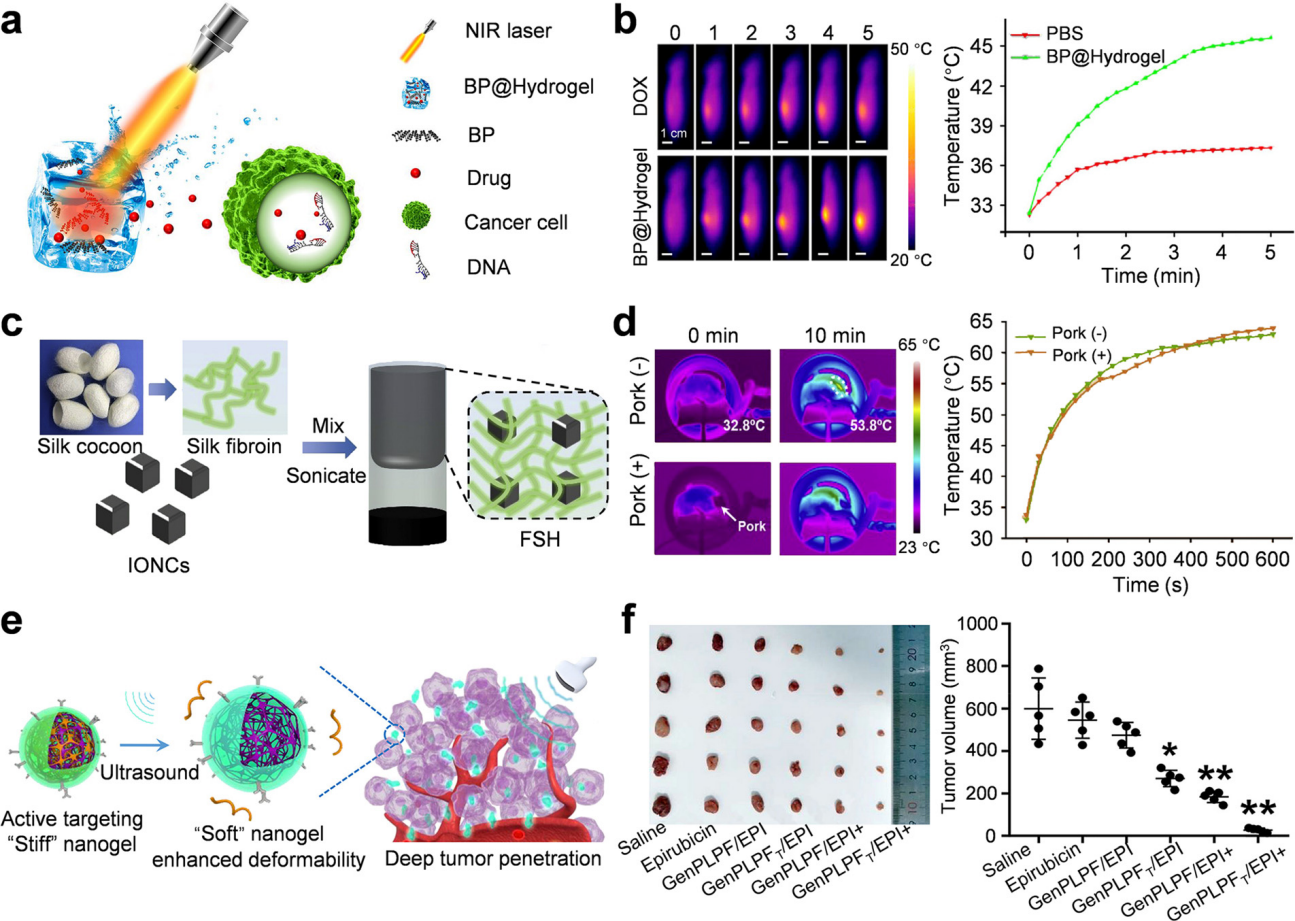
Stimuli-responsive biobased hydrogels. (a) Near-infrared irradiation causes the BP@hydrogel to release encapsulated drugs and kill cancer cells. (b) Real-time thermal imaging of mice and tumor temperature curves under 808 nm laser irradiation after injection of DOX and BP@hydrogel. (a) and (b) Adapted with permission from Qiu *et al.*, Proc. Natl. Acad. Sci. U. S. A **115**(3), 501–506 (2018). Copyright 2018, licensed under a PNAS license.^139^ (c) Preparation process of FSH; (d) infrared thermal imaging and temperature curves of FSH in mice with or without pig tissue coverage in magnetic field experiments. (c) and (d) Adapted with permission from Qian *et al.*, Biomaterials **259**, 120299 (2020). Copyright 2020 Elsevier.^118^ (e) GenPLPFT/EPI system is used for deep tumor therapy. (f) Photos and weights of mice tumors after 10 days of different treatment methods. (e) and (f) Adapted with permission from Sun *et al.*, ACS Nano **16**(6), 9183–9194 (2022). Copyright 2022 American Chemical Society.^119^

Hydrogels with photothermal response, which combine photothermal agents like polydopamine, gold nanoparticles, and MXene nanosheets, use light as a non-invasive trigger to achieve sol-gel transition and drug release. The light intensity can be adjusted, remotely controlled and has high spatial precision, making these materials very attractive in the fields of local therapy and regenerative medicine.^116,138^ Qiu *et al.* developed BP@hydrogel, which contains black phosphorus (BP) nanosheets with high photothermal conversion efficiency and low-melting agarose loaded with anti-cancer drugs [Fig. 6(a)].^139^ Once near-infrared light is irradiated, BP@hydrogel locally generates heat for thermotherapy. The light induces tumor ablation and melts the hydrogel to release the encapsulated drugs, achieving synergistic photothermal-chemotherapy effects [Fig. 6(b)].

Magnetic-responsive hydrogels encapsulate magnetic nanoparticles, such as iron oxide nanoparticles, which can achieve remote non-invasive control under an external magnetic field and are applied in the fields of targeted drug delivery, minimally invasive treatment, and tissue engineering.^117^ Qian *et al.* designed a ferrimagnetic silk fibroin hydrogel (FSH) composed of silk fibroin and iron oxide nanocubes [Fig. 6(c)].^118^ Under ultrasound guidance, FSH is locally injected into the deep tumor. Alternating magnetic fields generate local magnetothermal therapy [Fig. 6(d)]. The stable hydrogel structure has artery embolization effect, blocks tumor blood supply, and also extends to interventional embolization treatment. These findings indicate that magnetic-responsive hydrogels have clinical potential in deep and inoperable tumors.

Ultrasound is commonly used as a method of clinical imaging.^140^ Due to the non-invasive nature and ability to penetrate deep tissues, ultrasound has become a therapeutic trigger.^141^ Ultrasound-responsive hydrogels are designed to adjust their stability, elasticity, and permeability by acoustic stimulation to trigger the controlled release of encapsulated therapeutic agents. Sun *et al.* assembled Pluronic F127 and polylysine, and cross-linked with genipin to prepare an ultrasound-responsive peptide-based nanohydrogel (GenPLPFT) [Fig. 6(e)].^119^ Anti-ICAM-1 antibody is used for targeted tumor delivery. After external ultrasound exposure, the Pluronic F127 gel segment depolymerizes from the hydrogel, the structure expands and drug release is accelerated, thus enabling effective treatment of deep tumors [Fig. 6(f)].

## V. APPLICATIONS IN WOUND SEALING, HEMOSTASIS, AND TISSUE REPAIR

Rapid wound closure and efficient hemostasis are the key points in trauma, surgery, and emergency medicine. Uncontrolled bleeding is one of the main causes of death in surgical operations, severe trauma, and battlefield injuries. Biobased adhesive hydrogels have excellent biocompatibility, strong wet adhesion, and hemostatic activity and have great potential in these fields. Compared with traditional hemostatic agents and sutures, this hydrogel has the advantages of being able to conform to the shape of irregular wounds, reducing secondary damage to tissues, and being able to effectively penetrate in a bloody and changeable environment. The hemostatic effect of biobased hydrogels originates from the formation of physical barriers, biochemical blood clotting, and tissue adhesion. This part explores the potential mechanism of hemostasis, the design parameters of the materials used, and the conditions of different wounds.

### A. Mechanisms of wound sealing and hemostasis

**FIG. 7.**
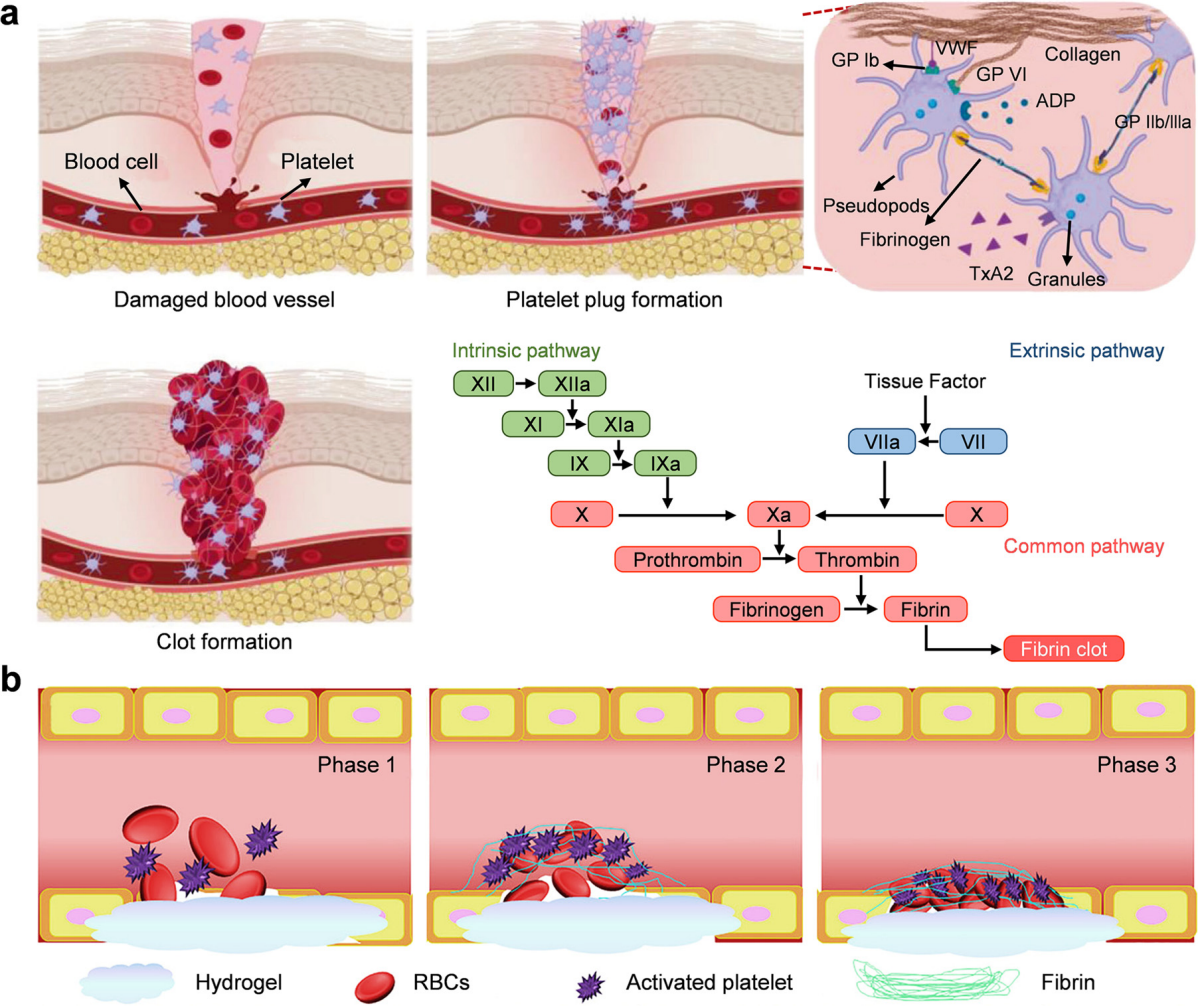
Hemostatic hydrogel for wound healing. (a) Primary hemostasis: vascular contraction followed by vWF-GP Ib and GPIIb/IIIa-fibrinogen interactions to promote platelet plug formation. Secondary hemostasis: The coagulation cascade is activated to form a stable fibrin network. Adapted with permission from Pourshahrestani *et al.*, Adv. Healthc. Mater. **9**(20), 2000905 (2020). Copyright 2020 the authors, published by Wiley-VCH GmbH, licensed under a Creative Commons Attribution (CC-BY-NC-ND) license.^142^ (b) Functional hydrogel accompanies time blood clot contraction during hemostasis. Adapted with permission from Guo *et al.*, Sci. Adv. **7**(29), eabf9635 (2021). Copyright 2021 The American Association for the Advancement of Science, licensed under a Creative Commons Attribution (CC-BY-NC) license.^151^

Hemostasis represents the first and most immediate phase of wound healing. It is rapidly initiated upon tissue injury and culminates in the formation of a stable hemostatic plug that halts bleeding. The hemostatic process consists of two principal stages—primary hemostasis (platelet plug formation) and secondary hemostasis (the coagulation cascade) [Fig. 7(a)].^142,143^ In primary hemostasis, vascular injury and endothelial rupture trigger vascular contraction and platelet activation. The initial vascular contraction squeezes the blood vessel to impede blood flow. Subsequently, the platelets adhere to the exposed subendothelial collagen through glycoprotein VI (GPVI) and integrin α2β1 receptors, and also bind von Willebrand factor (vWF) through the GP1b receptor.^144^ Adhesion activates platelets, changes their shape and secretes the contents of granules, such as serotonin, thromboxane A2 (TXA2), and adenosine diphosphate (ADP). These molecules change the conformation of the GPIIb/IIIa receptor, allowing fibrinogen to bind, and platelets aggregate to form a temporary hemostatic plug.^145,146^ This short-lived clot is the initial barrier to stop bleeding.

Secondary hemostasis is driven by the coagulation cascade. There are two interconnected pathways in this reaction—the intrinsic (contact activation) pathway and the extrinsic (tissue factor) pathway—ultimately converging to the common pathway to form a fibrin clot. In the intrinsic pathway, vascular injury generates a negatively charged surface to activate factor XII, and then, other coagulation factors are activated.^147^ The extrinsic pathway starts with tissue factor released from the damaged blood vessel wall. When Ca^2+^ appears, tissue factor combines with factor VII and activates factor VII. Both pathways converge by activating factor X. Factor X, along with its cofactor (activated factor V, namely, FVa) and Ca^2+^, combines to form the prothrombinase complex.^148^ This complex converts prothrombin to thrombin, which is a key serine protease that can convert fibrinogen to fibrin and also amplifies the steps of the coagulation cascade. Fibrin monomers polymerize into a fibrin network to enhance and stabilize the initial platelet plug. Activated factor XIII and Ca^2+^ cross-link the fibrin network into a strong clot.^149^

The intrinsic coagulation mechanism in the body relies on blood vessel constriction, platelet aggregation, and the coagulation cascade to plug the damage in small blood vessels. However, massive-pressure bleeding from major arteries and solid organs often exceeds the physiological hemostatic ability, posing a risk of serious injury.^127^ Therefore, the development of rapid, safe, and effective hemostatic pads is an urgent clinical need. Using biobased adhesive hydrogels for hemostasis is a complex, multimodal and synergistic thing, which can form a physical barrier and actively promote natural human blood clotting. Adhesive hydrogel-assisted hemostatic effects are often divided into passive and active, although they often play a role together.

Passive hemostasis relies on the formation of physical barriers and the absorption of fluids. Hydrogels rapidly absorb exudates and blood, concentrating platelets and coagulation factors at the wound. The barrier formed by hydrogels can not only stop bleeding but also stabilize blood clots. Porous and swellable polymer networks enhance the ability to absorb water and concentrate components, accelerating the formation of fibrin. Also, the hydration of hydrogels can maintain a moist environment, which is beneficial for cell migration and tissue regeneration. Hydrogels have biochemical interactions with blood components to achieve active hemostasis. Positively charged polymers rely on electrostatic attraction to negatively charged red blood cells and platelets, causing them to aggregate and activate. This mechanism mimics the role of natural coagulation cofactors and can initiate coagulation even without a complete coagulation cascade.^150^ Three-dimensional hydrogel matrices are good carriers for carrying bioactive substances and controlling their release. For example, a thrombin-containing hydrogel can achieve rapid injectable hemostasis through three steps [Fig. 7(b)]: (1) *In situ* polymerization of the hydrogel at the bleeding site forms an immediate physical seal; (2) the formed hydrogel matrix aggregates and activates platelets and red blood cells, and thrombin promotes the conversion of soluble fibrinogen to fibrin fibers, capturing more platelets; (3) platelets adhere to the fibrin network, induce blood clot contraction and stress generation, and form a mechanically stable structure to achieve the hemostasis purpose.^151^

### B. Application forms of adhesive hydrogels for wound sealing and hemostasis

The clinical performance of biobased adhesive hydrogels is related to their molecular composition and application form. These factors affect their operability, penetration behavior, and wound adaptability. The choice of application form mainly depends on the mechanical and rheological characteristics of the adhesive system. Shear modulus is very important for gelation dynamics and performance under specific conditions.^152^ Specifically, storage modulus (G′) is related to the elasticity of the adhesive matrix and cross-link density, and loss modulus (G″) reflects its viscous behavior. When G′ is greater than G″, the elastic component dominates, forming a solid-like gel network; when G′ is less than G″, the system is like a liquid precursor. The values of G′ and G″ can be accurately adjusted by adjusting cross-linking chemistry, polymer molecular weight, and network density.^153^ According to the shear modulus, tissue adhesives are divided into preformed type (G′ > G″) and *in situ* forming type (G′ < G″).

**FIG. 8.**
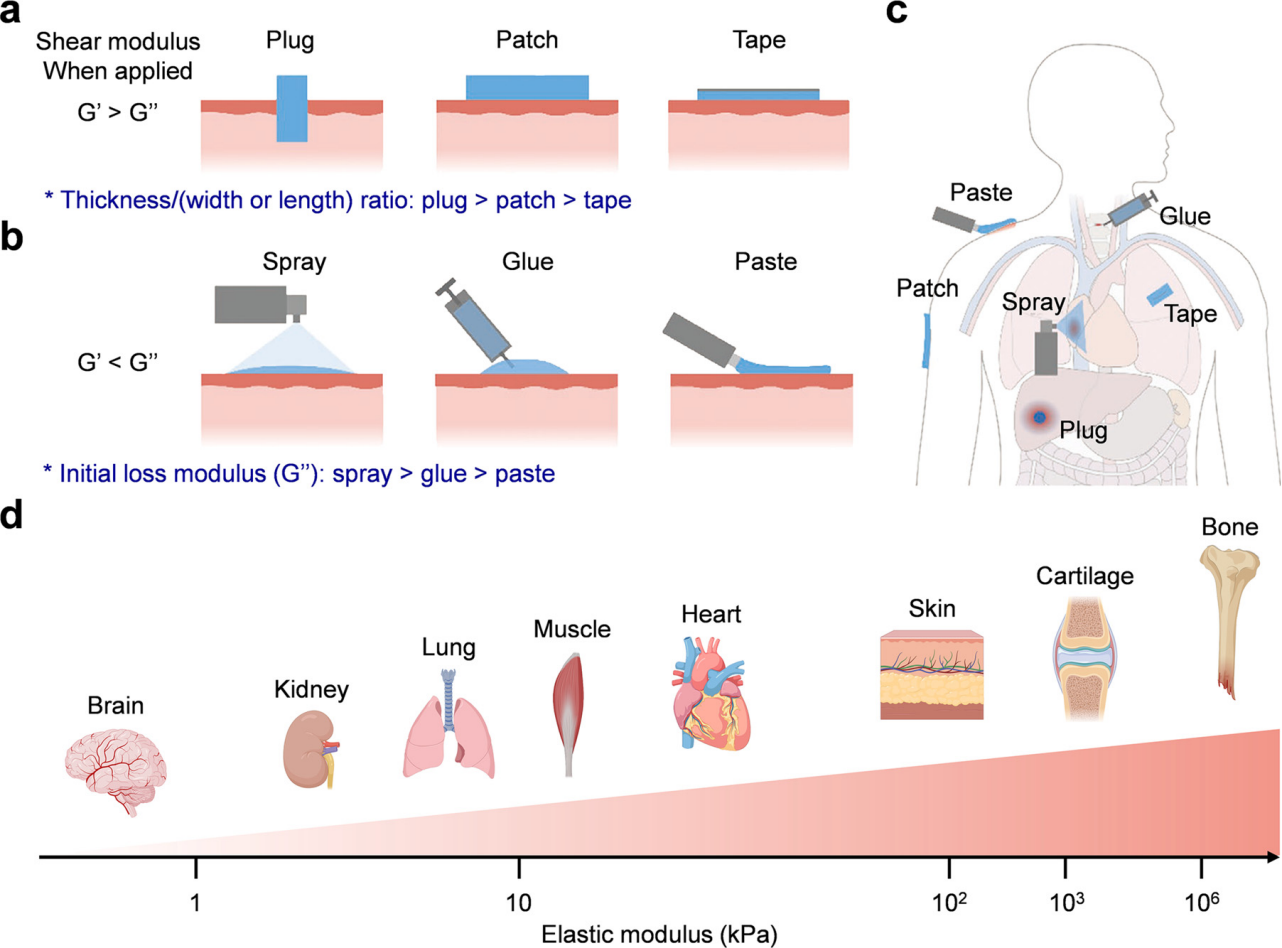
Application forms of adhesive hydrogels: (a) prefabricated solid adhesives, (b) *in situ* formed liquid adhesives, and (c) application scenarios of different adhesive hydrogels in tissues. (a)–(c) Adapted with permission from Ma *et al.*, Adv. Mater. **33**(24), 2007663 (2021). Copyright 2021 Wiley-VCH GmbH.^156^ (d) elastic moduli of several common biological tissues range from a few kilopascals to hundreds of megapascals, for example, the brain is about 0.5 kPa,^171^ the kidney is about 3 kPa,^171^ the lung is about 6 kPa,^170,171^ the muscle is about 10 kPa,^170,171^ the heart is about 20 kPa,^171^ the skin is about 100 kPa,^172^ the cartilage is about 1 MPa,^172^ and the bone is about 1 GPa.^172^ Created with Biorender.com.

Preformed adhesives with solid pads and adhesives with preformed geometric shapes are frequently used in clinical practice, especially in open surgery. They can be directly laminated on the tissue surface and molded to fill irregular defects.^53,127,154,155^ Due to their preformed geometric shapes, such adhesives come in various sizes and shapes, classified into plugs, patches, and tapes [Fig. 8(a)].^156^ Adhesive plugs are often designed as cylindrical and cuboid pads and implanted into tissue gaps/defects. The basic design criterion is to customize the geometric shape. Diameter and thickness are used to make up for the defects of sealing emergency wounds. The preformed plug has elastic sealing properties and can also stop bleeding, but the swelling rate needs to be controlled.^127^ When the hydrogel becomes overly expanded, it will squeeze the surrounding tissues.^157,158^ Adhesive patches containing hydrogels and elastomers are suitable for smooth areas like the skin and surgical sites. Because of strong adhesion, adjustable mechanical strength, and customizable functions, they are often used as dressings for chronic and large wounds. The adhesive tape belongs to thin sheets and is used to stick tissues and fix instruments to the skin. In wound management, sealing small incisions and fixing wearable devices to the skin is quite useful. Hard backings, such as PET film, can enhance mechanical strength and can also be routinely breathable. Prefabricated adhesives can make the structure uniform before use, but there are challenges when used in deep and internal wounds. Hydrogels with shape-memory and conversion characteristics may provide a promising way to overcome many limitations.^159–161^

*In situ* adhesives can well adapt to irregular and fragile tissues. When applied, they are in a liquid state (G′ < G″) and can also rapidly solidify into a gel in response to stimuli such as humidity, temperature, pH value, and light.^12,162–164^ Flow properties such as viscosity and shear thinning determine the application mode [Fig. 8(b)]. Low-viscosity formulations can be sprayed using compressed gas as an adhesive, which can quickly and evenly cover large and irregular surfaces.^165–167^ The spray design can be used as fast-controllable precursor deposition followed by *in situ* polymerization. To obtain a uniform and defect-free film, parameters such as spray distance and gas flow need to be optimized. However, problems like nozzle clogging and cleaning after use are challenges. Injectable adhesives are applied in minimally invasive surgeries with limited space and operation, such as laparoscopy.^168^ They can seal leaks and places unreachable by sutures. Cross-linking in the system is usually triggered by component mixing and external stimuli. Fast gelation may cause the applicator to block prematurely, and slow gelation has the risk of precursor diffusion from the target area. To balance these situations, a stimuli-triggered curing method is adopted. For example, ultraviolet light is started to be applied in the cross-linking process of poly(glycerol sebacate acrylate)-based surgical glue, and it can be bonded, hemostatic and sealed as required by being exposed for a few seconds.^169^

Organ tissue adhesives need to have mechanical matching with the target tissue [Fig. 8(c)].^125^ Elastic modulus reflects the ability of a material to resist stress-induced deformation and is a description of basic mechanical compatibility. The elastic modulus of biological tissues is usually between tens of pascals and hundreds of kilopascals [Fig. 8(d)].^170–173^ Many commercially available adhesives, such as cyanoacrylates, have a modulus in the range of hundreds of megapascals, which is much harder than the tissue being adhered.^170,174–176^ This mismatch will cause stress concentration at the tissue-adhesive interface, which may damage the adhesion effect, leading to delamination and tissue damage. These effects may also trigger inflammation and scarring. The adhesion modulus can be adjusted by polymer chemistry, cross-linking density, and molecular weight. Design strategies need to focus on reducing the mechanical difference between the adhesive and the target tissue so that the adhesion is more durable and biocompatible.

### C. Representative hemostatic applications for different wound types

**FIG. 9.**
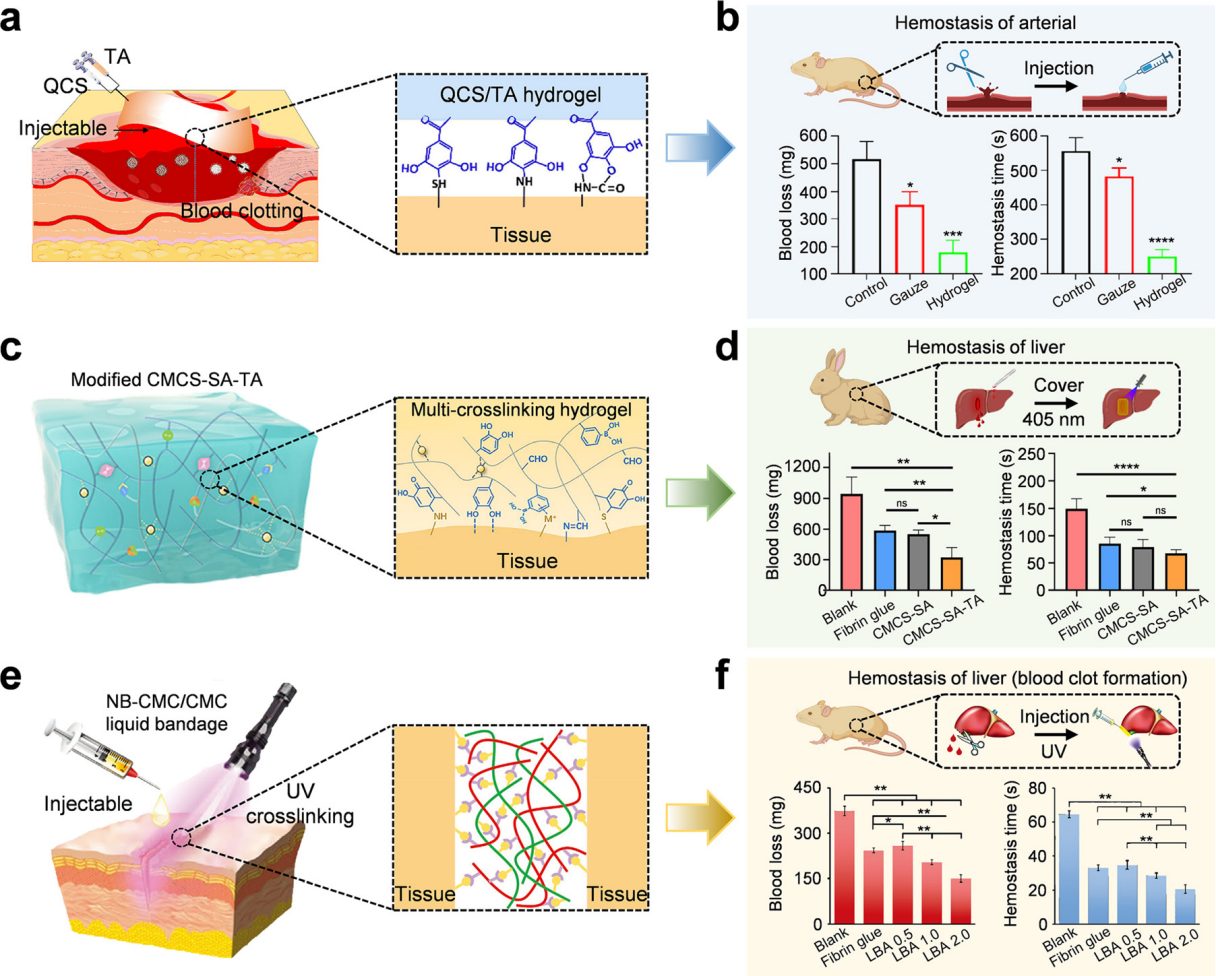
Adhesive hydrogels for hemostasis. (a) QCS/TA hydrogel has a superior hemostatic effect due to strong adhesion, which helps the wound to close quickly. (b) Schematic diagram of QCS/TA hydrogel for hemostasis of rat femoral artery, with quantitative blood loss and hemostasis time. ^*^p < 0.05, ^**^p < 0.01, ^***^p < 0.001, ^****^p < 0.0001. (a) and (b) Adapted with permission from Guo *et al.*, ACS Appl. Mater. Interfaces **14**(30), 34455–34469 (2022). Copyright 2022 American Chemical Society.^179^ (c) Schematic diagram of multi-scale cross-linked hydrogel–tissue adhesion. (d) Hemostatic properties of the hydrogel in a rabbit liver hemorrhage model, corresponding to the volume of blood loss and hemostasis time. (c) and (d) Adapted with permission from Zou *et al.*, Bioact. Mater. **16**, 388–402 (2022). Copyright 2022 the authors, published by Elsevier, licensed under a Creative Commons Attribution (CC-BY-NC-ND 4.0) license.^183^ (e) Ultraviolet exposure makes the amide cross-linking, and then the NB-CMC/CMC liquid bandage (LBA) is formed *in situ* for covalent binding and tissue adhesion. (f) Hemostasis with LBA is carried out using a rat liver hemorrhage model, and quantitative analysis is performed through blood loss and time. ^*^P < 0.05, ^**^P < 0.01, ^***^P < 0.001, ^****^P < 0.0001, ns: not significant. (e) and (f) Adapted with permission from Ma *et al.*, Adv. Funct. Mater. **30**(39), 2001820 (2020). Copyright 2020 Wiley-VCH GmbH.^188^

Massive bleeding is one of the main causes of death from trauma, especially in battlefields and emergency situations. Among civilians, more than half of traffic accident deaths are due to late and insufficient hemostasis.^177^ Transfusion after bleeding can cause secondary complications such as coagulopathy, infection, and multiple organ failure.^178^ Arterial spurting blood is difficult to handle because high blood pressure and fast blood flow can wash away temporary sealants. In such cases, adhesive hydrogels need to achieve immediate sealing and bonding under pulsatile blood flow of 120 mm Hg. When injured, immediate and rapid hemostasis can reduce blood loss and improve survival rate. Guo *et al.* mixed QCS with tannic acid (TA) to prepare an injectable, strongly adhesive hemostatic hydrogel [Fig. 9(a)].^179^ The QCS/TA hydrogel exhibited excellent adhesion, rapid self-healing, and biocompatibility *in vitro*. It can control arterial and deep non-compressive bleeding while significantly accelerating wound healing. In the femoral artery injury model, the blood loss in the hydrogel group was 179 mg, 517 mg in the control group, and 315 mg in the gauze group [Fig. 9(b)]. The hemostasis time was shortened to 250 s, which was much lower than 557 s in the control group and 483 s in the gauze group. Such effects come from the synergistic effect: (i) the protonated amino groups – ${\text{NH}}_{3}^{+}$ in CS adsorb negatively charged platelets and red blood cells, promoting aggregation and thrombus formation;^180,181^ (ii) tannic acid causes vasoconstriction and interacts with plasma proteins, enhancing clot stability.^182^

Fragile microvascular networks in organs such as the liver and spleen often have diffuse bleeding. Injuries require pads that can cover large irregular areas and also promote blood clotting on smooth tissues. Biobased hydrogels made from oxidized alginate, gelatin, and CS derivatives show obvious effects here. They rely on rapid ionic and covalent cross-linking to firmly adhere to moist tissues and conform to irregular shapes. For example, Zou *et al.* prepared a multi-cross-linked bioadhesive hydrogel by using carboxymethyl CS, sodium alginate, and TA with dynamic covalent bonds, photo-triggered covalent bonds, and hydrogen bonds [Fig. 9(c)].^183^ This hydrogel has good strength and toughness, and its adhesion strength reaches 1626 kPa, which is 12.3 times that of the commercial fibrin glue. In a rabbit liver injury model, the blood loss in the hydrogel group is half less than that in the fibrin glue group [Fig. 9(d)]. Fan *et al.* also carried out related research.^184^ It is reported that the injectable photocrosslinkable hydrogel based on dopamine-conjugated maleic HA is formed within 20 s under ultraviolet light. The catechol group of dopamine can make platelets and red blood cells aggregate and can also organize adhesion, thereby enhancing the hemostatic effect. Shin *et al.* developed the catechol-modified HA gel, and the liver adhesion of this gel is enhanced.^185^ Compared with the traditional photopolymerized HA gel, catechol-modified HA hydrogel improves the viability and function of hepatocytes. It exhibits strong wet adhesion and can stably transfer human hepatocytes to the asymmetric lobes of the mouse liver.

For patients with coagulation dysfunction, such as hemophilia, thrombocytopenia, and those receiving anticoagulant therapy, traditional hemostatic agents are ineffective, relying on the intrinsic coagulation cascade. At this time, biobased adhesive hydrogels that induce hemostasis through non-coagulation mechanisms are relatively important. Inspired by the intrinsic hemostatic and antibacterial properties of CS,^186,187^ Ma *et al.* developed a liquid bioadhesive (LBA) as a photoresponsive *in situ* cross-linked CS hydrogel [Fig. 9(e)].^188^ CMC is modified with o-nitrobenzyl alcohol to obtain NB-CMC, which is photolyzed under ultraviolet light to form aldehyde groups. The aldehyde groups react with tissue amine groups to form covalent bonds at the hydrogel–tissue interface, achieving rapid tissue integration and strong adhesion effects. It is worth noting that NB-CMC and CMC have positively charged amino groups, and they adsorb blood cells through electrostatic interaction, which enhances the formation of blood clots.^189,190^ During the operation, LBA adheres tightly to the wet liver incision and is also sealed under ultraviolet irradiation. The rapid sealing kinetics and high bonding strength are the keys to its excellent hemostasis [Fig. 9(f)]. The formation of blood clots and tissue sealing cooperate with each other, which makes LBA promising to be an effective hemostatic material for wound with coagulation disorders.

These progressions demonstrate that biobased adhesive hydrogels have super multi-functions in dealing with various bleeding conditions such as superficial abrasions to deep arterial and organ bleeding. Its *in situ* gelation, dynamic tissue adhesion, and inherent bioactivity enable rapid, durable, and biocompatible hemostasis under complex physiological conditions. Current research needs to further optimize gelation kinetics, mechanical strength, and biodegradability in order to expand its clinical applications as the next-generation surgical sealants and emergency hemostatic agents.

### D. Representative tissue repair applications in skin wound healing

Skin wound healing is a complex dynamic process, with stages of hemostasis, inflammation, proliferation, and remodeling. Biobased adhesive hydrogels assist these stages, providing a moist bioactive microenvironment that supports cell infiltration, angiogenesis, and re-epithelization. They have high water content to maintain tissue hydration, carry out gas exchange, prevent drying, and form a protective barrier to prevent infection. CS, HA, and gelatin-based hydrogels are effective because they have a structure similar to the extracellular matrix and inherent biocompatibility characteristics.

**FIG. 10.**
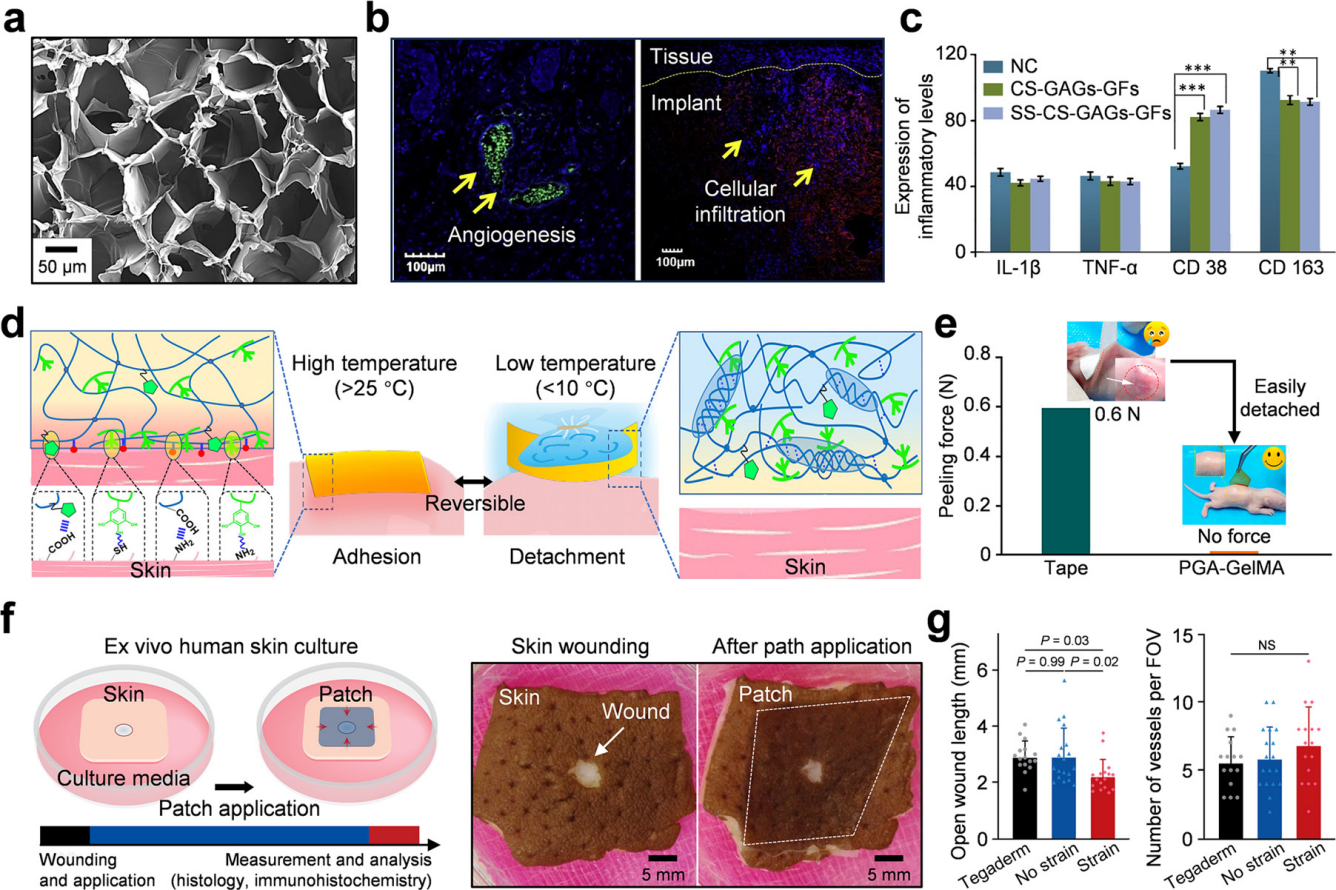
Adhesive hydrogel for skin wound healing. (a) SEM image shows that the SS–CS–GAGs hydrogel has an interconnected porous structure. (b) Confocal immunofluorescence analysis of blood vessels (the yellow arrows indicate blood vessels) and cell infiltration (the yellow arrows indicate infiltrating cells) in the hydrogel. (c) Experiment of the expression of cytokines TNF-α, IL-1β and macrophage markers CD38 (M1 type), CD163 (M2 type) 28 days after mouse skin implantation. ^**^P < 0.01, ^***^P < 0.001. (a)–(c) Adapted with permission from Sapru *et al.*, Carbohydr. Polym. **258**, 117717 (2021). Copyright 2021 Elsevier.^191^ (d) Adhesion and painless peeling of PGA-GelMA hydrogel when temperature changes: strong adhesion at body temperature, easy to remove after cooling. (e) Measure the peeling force of PGA-GelMA hydrogel and medical tape on the skin of newborn mice. The illustration presents the painless peeling of PGA-GelMA, but the tape removal causes skin damage. (d) and (e) Adapted with permission from Jiang *et al.*, ACS Nano **16**(6), 8662–8676 (2022). Copyright 2022 American Chemical Society.^198^ (f) The *in vitro* human skin culture model is used to evaluate the effect of strain-programmed hydrogel patches on diabetic wound healing, and representative pictures before and after patch application are shown. (g) The wound area and the number of blood vessels in each field of view on day 4 are quantified. Statistical analysis uses one-way ANOVA, followed by Tukey's multiple comparison test; ns: not significant. (f) and (g) Adapted with permission from Theocharidis *et al.*, Nat. Biomed. Eng. **6**(10), 1118–1133 (2022). Copyright 2022 the authors, under exclusive license to Springer Nature.^199^

Biobased hydrogels can act as bioactive reservoirs for controlled release of drugs, not only for physical property considerations. Smart hydrogel systems carry angiogenic and proliferative growth factors (GFs, such as vascular endothelial growth factor (VEGF), and epidermal growth factor (EGF)) and have great efficacy in accelerating epithelial regeneration and new blood vessel formation. For example, Sapru *et al.* developed a hydrogel based on silk sericin (SS), CS, and glycosaminoglycan (GAG), and also incorporated GFs [Fig. 10(a)].^191^ Scanning electron microscopy (SEM) presents a highly interconnected porous network with pore diameters of 55–145 *μ*m. The SS–CS–GAGs–GFs hydrogel has good biocompatibility and can effectively simulate the natural structure and biochemical signals of skin tissue. Immunofluorescence and hematoxylin–eosin (H&E) staining confirmed that in animals treated with the hydrogel, the maturity of blood vessels was improved and cells infiltrated into the surrounding dermal tissue [Fig. 10(b)]. At 28 days after implantation, the levels of pro-inflammatory cytokines TNF-α and IL-1β in the local skin tissue near the hydrogel were almost unchanged [Fig. 10(c)]. The SS–CS–GAGs–GFs hydrogel can also promote the polarization of anti-inflammatory macrophages and accelerates extracellular matrix remodeling and collagen deposition. These findings suggest the possibility of forming new tissues with low immune response, which is a promising approach for clinical treatment and regeneration of skin defects.

In addition to serving as bioactive scaffolds, adhesive hydrogels can act as protective barriers to cover wounds, absorb exudate, and accelerate healing. It makes surgery easier, shortens the recovery time, and improves the comfort of patients.^127,192^ However, highly adhesive hydrogels often need to be removed by mechanical debridement and surgery, which can cause pain and tissue damage.^193^ Therefore, adjustable viscosity is the key to application, especially in scenarios of reuse and long-term use.^194,195^ Degradable and easily removable adhesive hydrogels can reduce secondary damage during dressing changes.^196^ Chen *et al.* developed an injectable, quick-curing hydrogel that dissolves under cysteine-specific conditions.^197^ This hydrogel can carry out on-demand removal/replacement. For patients with fragile skin, such as preemies and diabetics, injury from using adhesive to remove is a major risk. As shown in Fig. 10(d), Lu *et al.* designed a skin-friendly adhesive patch composed of polymerized gallic acid (PGA) and GelMA, which has a thermo-responsive network sensitive to body temperature.^198^ When applied to the skin, the patch adheres firmly at physiological temperature and can be painlessly peeled off by cooling with an ice pack, and causes no damage to the delicate skin of newborn mice [Fig. 10(e)]. Zhao *et al.* also developed a hydrogel that can quickly adhere to moist wound tissues and self-contract to accelerate wound closure [Fig. 10(f)].^199^ This hydrogel realizes uniaxial and biaxial mechanical contraction and stress remodeling through water-induced recovery. The strain-programmable patches provide durable and detachable adhesiveness on diabetic wounds [Fig. 10(g)].

### E. Repair of specialized tissues

In addition to skin repair, biobased adhesive hydrogels can also be applied to regenerate various tissues, such as cartilage, bone, cardiac muscle, and mucosal systems. Through reasonable molecular design, they exhibit specific properties, such as mechanical strength, conductivity, and optical transparency, so as to fit the needs of specific tissues.

**FIG. 11.**
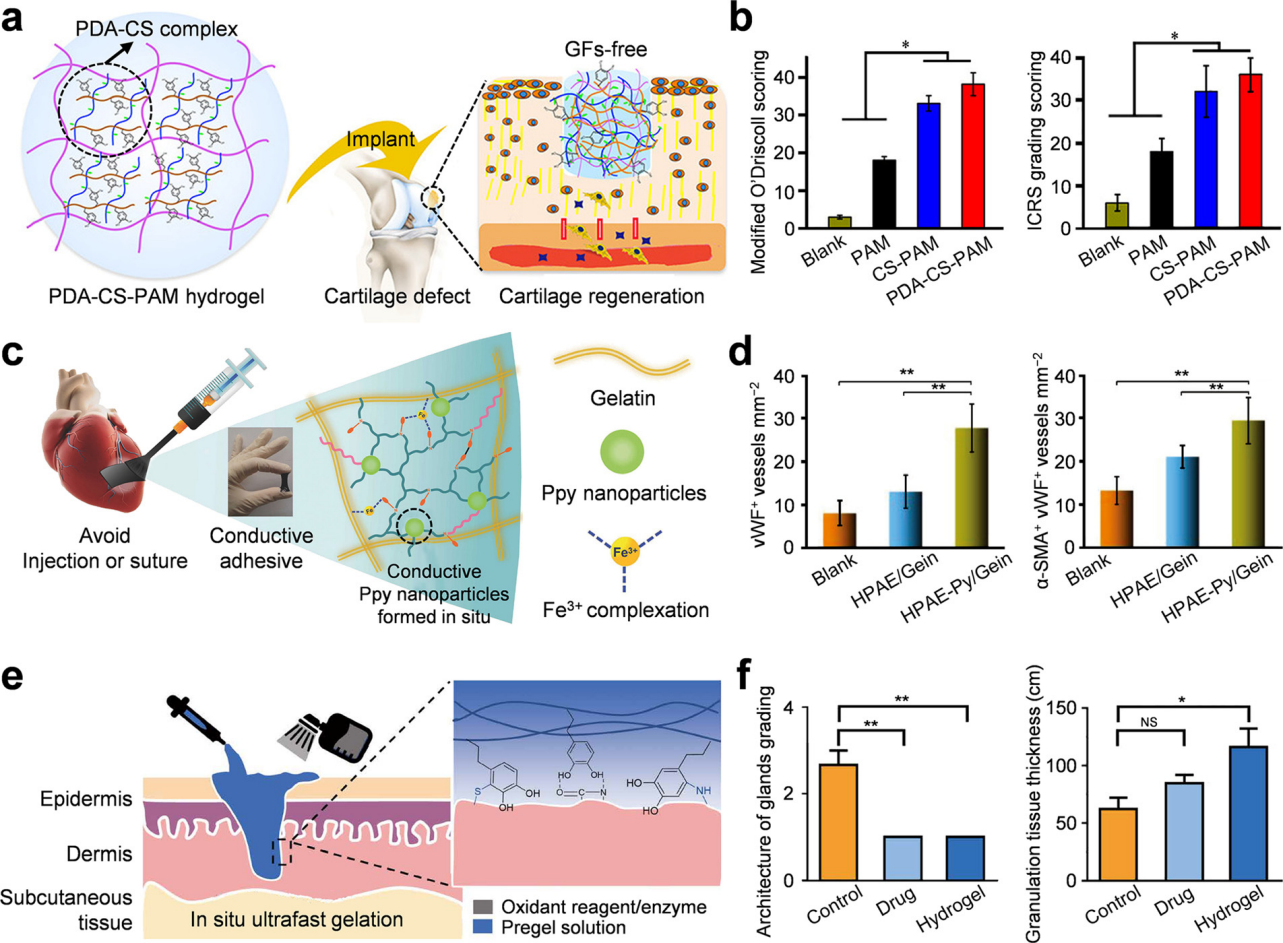
Adhesive hydrogels for tissue repair and regeneration. (a) Schematic of PDA–CS–PAM hydrogel formation and its application in cartilage defect repair. (b) Quantitative scoring of regenerated cartilage using Cartilage Repair Society (ICRS) and modified O'Driscoll systems. (a) and (b) Adapted with permission from Han *et al.*, ACS Appl. Mater. Interfaces **10**(33), 28015–28026 (2018). Copyright 2018 American Chemical Society.^205^ (c) Schematic of conductive adhesive HPAE-Py/Geln hydrogel and its application for myocardial repair by directly applying it to rat heart tissue. (d) Quantify the vWF-positive and α-SMA + vWF double-positive blood vessels in the 4-week-old infarct area; ^**^p < 0.01. (c) and (d) Adapted with permission from Liang *et al.*, Adv. Mater. **30**(23), 1704235 (2018). Copyright 2018 Wiley-VCH Verlag GmbH & Co. KGaA, Weinheim.^214^ (e) Schematic of *in situ* formation of HA–CAT–NCSN hydrogel in the stomach. (f) Evaluate gastric slices at day 14 after the application of HA–CAT–NCSN hydrogel and sucralfate, and perform analysis according to gland structure and granulation tissue thickness. Statistical significance analyzed by one-way ANOVA, followed by Tukey's multiple comparison test. ^*^P < 0.05, ^**^P < 0.01, ns: not significant. (e) and (f) Adapted with permission from Xu *et al.*, Sci. Transl. Med. **12**(558), eaba8014 (2020). Copyright 2020 The American Association for the Advancement of Science.^89^

In regenerative medicine, the self-repair ability of cartilage and bone is limited and the environment is complex. At present, clinical methods for articular cartilage defect, such as microfracture, cell transplantation, and tissue transplantation, will encounter problems such as few donor sources, low efficiency of chondrocytes, and poor integration with surrounding tissues.^200^ Biobased hydrogels show great potential because they can adjust mechanical properties and possess bone and cartilage bioactivity.^201^ Hydrogels made of collagen,^202^ HA,^203^ and chondroitin sulfate^204^ simulate the components and viscoelasticity of natural extracellular matrix of chondrocytes and can provide lubrication, integration support, promote proliferation of chondrocytes, and deposition of extracellular matrix. Han *et al.* developed a tissue-adhesive hydrogel composed of polydopamine–chondroitin sulfate–polyacrylamide (PDA–CS–PAM) for articular cartilage engineering [Fig. 11(a)].^205^ This hydrogel constructs a biomimetic microenvironment for chondrocytes, enabling them to adhere, proliferate, and anchor to the tissue. Without the addition of extra growth factors, the PDA–CS–PAM hydrogel helps enhance cartilage repair, and it performs better than the control group and the group using only PAM in terms of naked eye observation and histological scoring [Fig. 11(b)]. Adhesive hydrogels, in addition to encapsulating autologous chondrocytes, can also contain bioactive components and promote cartilage formation with external stimuli such as pulsed electromagnetic fields.^206–208^ Studies have shown that adjusting the composition of biobased adhesive molecules can produce hydrogels with mechanical and biological functions for the musculoskeletal field. However, when maintaining the cartilage protection function, obtaining higher mechanical strength, wear resistance, and good friction performance is a major challenge for cartilage regeneration.

The repair of myocardial infarction requires that the material be firmly adhered to the contracting heart to maintain mechanical and electrical coupling.^209^ Conductive biobased hydrogels have attracted more attention because they can not only enhance the damaged myocardium but also restore conductivity. Cardiac hydrogels need to combine strong wet adhesiveness, high stretch, and fatigue resistance to adapt to long-term cyclic strain.^210^ The regenerative ability of the heart is low, thus integrating drug therapy and electrical stimulation therapy in adhesive hydrogels is a promising method to enhance myocardial regeneration.^211,212^ Natural materials such as silk fibroin, gelatin, and HA are often used because of their biocompatible elasticity, and conductive components such as MXene, polypyrrole, and graphene oxide provide electrical functions for cardiac conduction.^213^ Liang *et al.* developed a brush-coated conductive hydrogel to make a therapeutic cardiac patch for inhibiting myocardial infarction and promoting electrical signal conduction after injury [Fig. 11(c)].^214^ This hydrogel HPAE-Py/Geln is synthesized by the copolymerization of pyrene and dopamine in a hyperbranched amine-epoxy network induced by Fe^3+^. The obtained hydrogel has strong wet adhesion and can be easily applied on the infarcted myocardium to form a tight and non-leaking seal. Post-treatment analysis found that cardiac function recovery was associated with inhibited infarct size [Fig. 11(d)]. This group subsequently prepared an adhesive hydrogel loaded with adipose-derived stem cells (ADSCs) to enhance cardiac repair.^215^ The adhesive hydrogel can restore cardiac function through hemostasis, mechanical support, and myocardial regeneration. However, current research mostly focuses on small puncture injuries, and solving large myocardial defects is a key challenge.

Biobased hydrogels are often used in fragile mucosal tissues. For example, in the gastrointestinal tract and cornea, they need to have flexibility, transparency, and pH responsiveness. The gastrointestinal tract is the place for food transportation, digestion, and absorption, and needs to deal with peristalsis and acidic secretions. Hydrogels used in this environment need to have mechanical adaptability and chemical stability.^216^ Adhesive hydrogels have good sealability, can be used alone and as an auxiliary means for suturing, can prevent leakage, and accelerate ulcer repair.^89,217^ Due to the limitations of gastrointestinal delivery, such hydrogels need to be injectable and self-healing for minimally invasive endoscopic drug administration.^218^ Their wet adhesion properties must can adhere to irregular surfaces and also resist digestive juices.^82^ The pH value in the stomach is extreme and fluctuating. The hydrogels need to gel quickly in an acidic environment and adhere durably.^218,219^ As shown in Fig. 11(e), Xu *et al.* prepared a pH-independent, ultra-fast-gelling HA–catechol–thiourea hydrogel (HA–CAT–NCSN).^89^ The endoscope delivers the hydrogel into the pig's stomach, where it forms a hydrogel *in situ* and adheres to the ulcer within 48 h. NCSN–CAT crosslinks on the moist acidic tissue and has strong adhesion ability. Compared with the standard drug sucralfate, the HA–CAT–NCSN hydrogel can accelerate ulcer healing and epithelial regeneration [Fig. 11(f)].

In clinical practice, common methods for corneal repair include keratoplasty, endothelial transplantation, and commercial tissue sealants.^209,220^ During the corneal repair process, maintaining optical transparency and oxygen permeability is the key to visual recovery. HA-, GelMA-, and fibrin-based hydrogels have an optical transparency of more than 90% and can form stable, reversible and non-cytotoxic bonds with the corneal stroma.^163,221,222^ In particular, the photocurable HA-based adhesive can quickly seal corneal perforations within seconds while maintaining transparency and causing less irritation to the eyes.^221^ However, limited control over cross-linking kinetics and insufficient biomechanical stability is a key obstacle to clinical transformation. These bioadhesives can act as alternatives to sutureless ophthalmic surgery and reduce postoperative complications.

In summary, biobased adhesive hydrogels have properties such as adhesion and biocompatibility, and are applied in tissue repair and skin wounds. They can moisturize, regulate inflammation, and promote epithelial and vascular regeneration. When repairing bone and cartilage defects, they can provide bone induction conditions and mechanical support. In heart tissue repair, they can restore conductivity and structural integrity. At the mucosal injury site, they can gently and durably adhere by virtue of biocompatibility. With the development of molecular design, scalable manufacturing, and regulatory assessment, biobased adhesive hydrogels are expected to move from laboratory innovation to clinical feasible biomaterials.

## VI. CONCLUSIONS AND FUTURE PERSPECTIVES

Biobased adhesive hydrogels represent a new generation of biomedical materials. Through precise molecular design and structural regulation, they have great potential in wound management and tissue repair fields. This review summarizes its design strategies, adhesion mechanisms, functional properties, and applications in hemostasis and tissue regeneration. Studies show that natural polymer-based hydrogels have good biocompatibility and degradability. Through functional modification, they can achieve strong adhesion to moist tissues, controlled drug release, and specific biological functions. These materials simulate the structural functions of the extracellular matrix and provide an ideal microenvironment for cell proliferation, differentiation, and tissue regeneration. Current research shows that with a reasonable selection of materials and structural design, biobased adhesive hydrogels can meet different needs from skin wounds to specific tissue repair scenarios, such as bone, heart tissue, and mucosa. Although there has been progress, there are still key challenges for biobased adhesive hydrogels to be widely applied in clinical applications. Future development requires close integration of multiple disciplines to bridge the gap between laboratory design and clinical application. The next-generation clinically feasible biological adhesives need to have high stickiness, safety, controllable interaction of active groups, long-term biological stability, and predictable degradation kinetics under pathological conditions. The ultimate goal is to create an intelligent, adaptive, and patient-specific hydrogel system that can integrate with the body to carry out personalized medicine.

### A. Key challenges

Adhesive hydrogels based on developmental biology need to balance strong stickiness and tissue safety.^223^ Irreversible covalent bonds can firmly seal and stop bleeding. However, excessive stickiness may cause secondary tissue damage when removing dressings, which is particularly obvious in delicate tissues such as skin, mucosa, and cornea. Future research needs to explore controllable and reversible adhesion mechanisms without causing mechanical damage. Strategies based on stimuli response and dynamic covalent chemistry seem very promising. Adjust the adhesion strength by designing materials, go through the healing stage, minimize tissue damage, and maintain functional integrity.

Another key challenge is that adhesive hydrogels need to adapt to the environment of dynamic tissues such as the heart, lungs, and joints, where stress can lead to separation.^224^ Hydrogels have viscoelasticity that simulates the mechanical properties of natural tissues, as well as self-healing and energy-dissipating structures, which are important for adhesion under physiological movement. Incorporating nanofillers, double-network structures, and dynamic cross-linking can improve fatigue resistance and mechanical toughness. Real-time *in vivo* imaging and computational modeling may provide deeper insights into the evolution of adhesive interfaces under mechanical loading, thus guiding the rational design of hydrogels that can withstand movement.

Despite encouraging preclinical results, many adhesive hydrogels fail to advance beyond animal models due to multiple translational barriers. One major limitation lies in the limited predictive value of commonly used animal models, which often do not fully recapitulate human tissue complexity, scale, and mechanical dynamics. Adhesive performance observed in small-animal models may not translate to larger, more mechanically demanding human tissues. Manufacturing and scalability present additional challenges. Many advanced adhesive hydrogels rely on complex synthesis routes, precise component ratios, or *in situ* cross-linking reactions that are difficult to reproduce under good manufacturing practice (GMP) conditions. Sterilization processes may further alter chemical functionality or mechanical properties, compromising performance. Regulatory and safety considerations also hinder clinical translation. Reactive chemistries, such as aldehyde or catechol groups, raise concerns regarding long-term biocompatibility, toxicity, and degradation by-products, requiring extensive validation. Moreover, the absence of standardized regulatory pathways and clinically relevant benchmarks for tissue adhesives complicates approval and adoption. Finally, limited large-animal and human clinical studies restrict confidence in long-term efficacy and safety. Bridging this gap requires early integration of translational considerations, standardized evaluation frameworks, and closer collaboration between materials scientists, clinicians, and regulatory experts.

Finally, minimally invasive surgical techniques, including laparoscopic, endoscopic, and catheter-assisted procedures, have become increasingly important in modern clinical practice due to reduced trauma, faster recovery, and lower complication rates. In this context, adhesive materials designed for minimally invasive applications must meet distinct requirements, such as injectability through narrow delivery channels, rapid *in situ* gelation, and reliable adhesion under wet and confined environments. Recent studies have demonstrated that biobased adhesive hydrogels can be adapted for minimally invasive adhesion by tailoring their rheological properties, gelation kinetics, and interfacial chemistry, enabling localized tissue sealing and hemostasis without extensive suturing. Looking forward, further integration of adhesive material design with minimally invasive surgical techniques, along with improved delivery strategies and clinical validation, is expected to expand the practical utility of biobased adhesives in minimally invasive tissue repair.

### B. Next-generation biobased adhesive hydrogels

Next-generation biobased adhesive hydrogels may focus on personalization, functional integration, and mechanical understanding. First, the morphology of hydrogels customized for patients in personalized and precision medicine involves wound type, tissue composition, and healing kinetics. For example, technologies such as 3D bioprinting and microfluidic synthesis can fabricate hydrogels with precisely controllable composition, stiffness, and degradation rate. These intelligent adhesives can dynamically match the biochemical and mechanical properties of specific tissues of patients. In addition, a key direction is to develop integrated treatment systems that integrate diagnosis, treatment, and monitoring in one hydrogel platform. Integrated bioadhesives may contain biosensors for real-time detection of infections, pH values, and temperatures, and can also release responsive drugs and growth factors to form a closed-loop healing system. Integrating tissue adhesion, therapeutic delivery, and biological signal feedback, smart hydrogels can automatically adjust their properties according to wound conditions, reducing clinical interventions and improving treatment precision. The mechanisms of the interaction between the extracellular matrix and tissues at the molecular and cellular levels need to be deeply understood. Traditional animal models provide valuable insights, but it is often difficult to simulate the complex physiological functions of humans. Advanced *ex vivo* platforms, such as organoids, organ-on-a-chip systems, and microfluidic wound models, provide unprecedented opportunities to study adhesion dynamics, immunomodulation, and regeneration in a biomimetic environment.

The innovation of materials and the integration of biological insights will generate intelligent, self-adaptive, and safe biobased adhesive hydrogels. These adhesive hydrogels can not only act as passive wound dressings, but also as biological interaction interfaces to sense physiological changes and coordinate healing in a controllable and patient-specific manner. With continuous interdisciplinary research, biobased adhesive hydrogels are expected to redefine wound management and tissue repair, bringing transformative solutions for personalized and intelligent medicine in the near future.

## Data Availability

Data sharing is not applicable to this article as no new data were created or analyzed in this study.
